# More Specific Signal Detection in Functional Magnetic Resonance Imaging by False Discovery Rate Control for Hierarchically Structured Systems of Hypotheses

**DOI:** 10.1371/journal.pone.0149016

**Published:** 2016-02-25

**Authors:** Konstantin Schildknecht, Karsten Tabelow, Thorsten Dickhaus

**Affiliations:** 1 Weierstrass Institute for Applied Analysis and Stochastics, Berlin, Germany; 2 Institute for Statistics, University of Bremen, Bremen, Germany; University of Texas at Austin, UNITED STATES

## Abstract

Signal detection in functional magnetic resonance imaging (fMRI) inherently involves the problem of testing a large number of hypotheses. A popular strategy to address this multiplicity is the control of the false discovery rate (FDR). In this work we consider the case where prior knowledge is available to partition the set of all hypotheses into disjoint subsets or families, e. g., by a-priori knowledge on the functionality of certain regions of interest. If the proportion of true null hypotheses differs between families, this structural information can be used to increase statistical power. We propose a two-stage multiple test procedure which first excludes those families from the analysis for which there is no strong evidence for containing true alternatives. We show control of the family-wise error rate at this first stage of testing. Then, at the second stage, we proceed to test the hypotheses within each non-excluded family and obtain asymptotic control of the FDR within each family at this second stage. Our main mathematical result is that this two-stage strategy implies asymptotic control of the FDR with respect to all hypotheses. In simulations we demonstrate the increased power of this new procedure in comparison with established procedures in situations with highly unbalanced families. Finally, we apply the proposed method to simulated and to real fMRI data.

## Introduction

Modern research is increasingly concerned with large-scale experiments and complex experimental designs. From a statistical perspective the analysis of such experiments often involves the issue of multiple testing of a large number (say *m*) of individual hypotheses. The development of methods to deal with this issue is a very active field of research with many sophisticated procedures emerging, e. g., taking a specific structure in the set of hypotheses into account; see, for example, Sections 3.3 and 12.2 in [1].

One example is the analysis of functional magnetic resonance imaging (fMRI) data; see [2] for an overview. At each unit of measurement (voxel) on a regular grid a statistical test is to be performed for the null hypothesis of no activation versus the alternative hypothesis of activation of the voxel (a signal detection problem). In such an application, the number *m* is often of the order of magnitude of several hundreds of thousand hypotheses.

The family-wise error rate (FWER) and the false discovery rate (FDR) are two established notions for measuring the type I error of a multiple test. The FWER denotes the probability of at least one false rejection among the *m* individual tests, and a multiple test is said to control the FWER (in the strong sense), if the latter probability is bounded by a pre-defined significance level *α* over the whole parameter set of the statistical model. One simple way to control the FWER is to carry out every individual test at the adjusted level *α*/*m*, commonly referred to as the Bonferroni correction. However, this ignores the spatial correlations of the data (cf. [3]), and can often be improved by multivariate methods. Another strategy for fMRI signal detection with FWER control incorporating the spatial dependencies of the hypotheses is based on the geometry of random fields, see [4] and [5].

On the contrary, the FDR is defined as the expected proportion of type I errors among all rejections of the multiple test *φ*, and *φ* is said to control the FDR at a given level *α* ∈ (0, 1) if this expected proportion is smaller than *α* for all parameter values of the considered statistical model. Applying this criterion leads to more liberal multiple tests, meaning that on average more null hypotheses can be rejected. The Benjamini-Hochberg procedure (or linear step-up (LSU) test *φ*^*LSU*^, see [6]) for FDR control has become very popular in fMRI research, cf. [7]. Meanwhile, FDR control is an established criterion for the analysis of high-dimensional data, and is agreed upon to provide a suitable interpretation of the results.

When structural information regarding the hypotheses is at hand, it is often possible to incorporate this external knowledge into the statistical methodology in order to improve the test procedures with respect to power or specificity. In the fMRI context, weighted variants of *φ*^*LSU*^ considered in previous work incorporate different aspects of the spatial structure of the activation areas, which are typically organized as clusters of activation rather than as singular spots. Furthermore, the functional organization of the brain defines specific regions of interest related to specific functions that are accessible by suitable experimental paradigms, see [8]. A very old example for such a functional atlas based on cytoarchitecture is the Brodmann atlas (cf. [9]). Clustering techniques to define regions of interest and to incorporate the (in general) heterogeneous cluster sizes into *φ*^*LSU*^ were employed in [10] and [11]. Relatedly, in [12] and [13] a case was studied in which the set of hypotheses can be divided into disjoint groups with potentially different proportions of activated voxels by means of a-priori knowledge. The authors demonstrated higher power of their proposed weighted *φ*^*LSU*^ tests in comparison with the standard LSU procedure if the fraction of true null hypotheses differs between the groups.

Another class of weighted FDR-controlling multiple tests introduces a second layer of hypotheses which are added to the original set of the *m* individual hypotheses. Namely, each of the considered disjoint groups is associated with the group-specific null hypothesis of no activation of the whole group. This leads to a hierarchical hypotheses structure with two levels. One level consists of all the group hypotheses and the other of all the *m* individual hypotheses. In such a context, hierarchical multiple test procedures consist of two stages: First, the group hypotheses are tested, and families for which the group hypothesis cannot be rejected are excluded from the analysis. This strategy relaxes the (remaining) multiplicity for the second stage, where the individual hypotheses are tested. This situation was investigated, for instance, in [14, 15] and [16], and is also often encountered in other application fields like genetic association studies (cf. [17]), gene expression analyses (cf. [18]), or in electroencephalography research (cf. [19]).

In this paper we develop a new two-stage method for FDR control in the fMRI context that takes into account an a-priori partition of the brain into disjoint families of voxels. The main innovation is that non-linear critical values or rejection curves, respectively, are utilized in the second stage. To this end, we make use of the approach in [20] and [21] for implicit adaptation of FDR-controlling multiple test procedures to the amount of signals. While these papers only considered the individual hypotheses, we apply their reasoning within every group which is still under consideration in the second stage of the hierarchical two-stage test. This leads to high sensitivity regarding the voxels within such a group. This is combined with a Bonferroni-type multiplicity adjustment in the first stage, implying a good specificity during the detection of active regions (testing of the group hypotheses). We prove that this procedure controls the FWER on the set of the family hypotheses, as well as, asymptotically as *m* → ∞, the FDR within each family and the global FDR (gFDR), which is the FDR with respect to all individual hypotheses.

The remaining sections are structured as follows. In Section “Methods” the mathematical notation is set up, some known results about FDR control are reported, the considered two-stage procedures are introduced and their statistical properties are analyzed. To evaluate the proposed new procedure we perform a number of simulations, and we analyze real fMRI data. To this end, the experimental setups and the most important results are explained and reported in Section “Results”. We conclude with a discussion in the subsequent Section “Discussion”. Lengthy mathematical derivations are deferred to S1 Appendix. For the sake of completeness, additional experimental results are provided in S1 and S2 Tables as well as in the figures in S1, S2, S3, S4, S5, S6 and S7 Figs.

## Methods

### Notation and preliminaries

We denote the number of families of hypotheses by *k* and the families themselves by $H_{1},\ldots ,H_{k}$. Each set $H_{\ell }$ is assumed to consist of *m*_*ℓ*_ > 0 individual hypotheses *H*_*ℓ*1_, …, *H*_*ℓm*_*ℓ*__, 1 ≤ *ℓ* ≤ *k*. In addition, for each of the *k* groups we consider a screening (or family) hypothesis $H_{\ell }^{f},1\leq \ell \leq k$, which we will formally define in Definition 4. The aims of the statistical analysis are (i) FDR control in each family $H_{\ell }$ separately, (ii) FDR control with respect to all individual hypotheses pooled together, denoted by the global FDR, (iii) FWER control on the group level, i. e., with respect to ${\left(H_{\ell }^{f}\right)}_{1\leq \ell \leq k}$. We assume that for each hypothesis a (marginal) *p*-value is available, which we identify by the same sub- and / or superscript as the corresponding hypothesis.

**Definition 1 (Linear step-up test *φ*^*LSU*^)** *Denote by* *p*_1:*m*_ ≤ *p*_2:*m*_ ≤ … ≤ *p*_*m*:*m*_ *the ordered* *p*-*values for a collection* $H_{m}=\left\{H_{i},i\in I=\left\{1,\ldots ,m\right\}\right\}$ *of null hypotheses at hand. Furthermore, let* *H*_1:*m*_, …, *H*_*m*:*m*_ *denote the re-ordered null hypotheses in* $H_{m}$, *according to the ordering of the* *p*-*values. Then, the linear step-up test* *φ*^*LSU*^ *at FDR level* *α* ∈ (0, 1) *rejects exactly the hypotheses* *H*_1:*m*_, …, *H*_i*:*m*_, *where*

$$i^{*}=\max\left\{i\in I:\;p_{i:m}\leq i\alpha /m\right\}.$$ (1)

*If the maximum in* Eq (1) *does not exist, then no hypothesis is rejected*.

The linear step-up test belongs to the broad class of step-up-down (SUD) multiple tests, introduced in [22].

**Definition 2 (Step-up-down test of order λ in terms of *p*-values, cf. [21])** *Let* *p*_1:*m*_ ≤ *p*_2:*m*_ ≤ … ≤ *p*_*m*:*m*_ *and* *α* *be defined as in Definition 1. For a tuning parameter* λ∈{1,…,m} *a step-up-down test* *φ*^λ^ = (*φ*_1_, …, *φ*_*m*_) *(say) of order* λ *based on some critical values* *α*_1:*m*_ ≤ ⋯ ≤ *α*_*m*:*m*_ *is defined as follows: If* *p*_λ:*m*_ ≤ *α*_λ:*m*_, *set* i* = max{*j* ∈ {λ, …, *m*}:*p*_*i*:*m*_ ≤ *α*_*i*:*m*_ *for all i* ∈ {λ, …, *j*}}, *whereas for* *p*_λ:*m*_ > *α*_λ:*m*_, *put* i* = sup{*j* ∈ {1, …, λ − 1}:*p*_*j*:*m*_ ≤ *α*_*j*:*m*_} (sup∅ = −∞). *Define* *φ*_*i*_ = 1 *if* *p*_*i*_ ≤ *α*_i*:*m*_ *and* *φ*_*i*_ = 0 *otherwise* (*α*_−∞:*m*_ = −∞).

*A step-up-down test of order* λ = 1 *or* λ = *m*, *respectively, is called step-down (SD) or step-up (SU) test, respectively. If all critical values are identical, we obtain a single-step test*.

In case of *φ*^*LSU*^, λ = *m* and *α*_*i*:*m*_ = *iα*/*m* for all 1 ≤ *i* ≤ *m*. In general, the choice of the order λ and of the critical values employed in an SUD test for FDR control depends on model assumptions; cf. Table 5.1 in [1].

**Definition 3 (AORC-based critical values, cf. [20] and [21])** *Under the assumptions of Definitions 1 and 2, we denote by* $\varpi_{\lambda }^{AORC}$ *the SUD test with critical values*

$$\alpha_{i:m}=\frac{i\alpha }{m-i(1-\alpha )},\;1\leq i\leq m.$$ (2)

The critical values in Eq (2) correspond to the so-called asymptotically optimal rejection curve (AORC) introduced in [20]. For suitable choices of λ and under the assumption of stochastically independent *p*-values, $\varpi_{\lambda }^{AORC}$ has been shown to exhaust the FDR level *α* asymptotically as *m* → ∞, while *φ*^*LSU*^ is not exhausting *α* if the number of true null hypotheses is smaller than *m*.

In a two level situation with group hypotheses and individual hypotheses, a two-stage procedure can be employed. In our case we are interested in testing the hypotheses within a family $H_{\ell }$ only if this family has been declared active, meaning that $H_{\ell }^{f}$ has been rejected in the first stage of analysis. In the fMRI context a family consists of many individual hypotheses and we consider a single activation in a family (an isolated signal) rather as noise than as evidence for activation of the family. Therefore we employ a criterion which defines a family as active if there is at least a certain proportion of activated voxels in the family. This proportion has to be predefined in advance. A useful tool to formalize activity of families in this context is the partial conjunction hypothesis introduced in [23].

**Definition 4** *For a given integer* 1 ≤ *u*_*ℓ*_ ≤ *m*_*ℓ*_, *the* *u*-*partial conjunction hypothesis* $H^{u_{\ell }/m_{\ell }}$ *for family* $H_{\ell }$ *is defined as the set of parameters such that* $H_{\ell }$ *contains less than* *u*_*ℓ*_ *false null hypotheses, with corresponding alternative given by the set of parameters such that the number of true alternatives in* $H_{\ell }$ *is at least equal to* *u*_*ℓ*_. *Based on this, we let* $H_{\ell }^{f}=H^{u_{\ell }/m_{\ell }}$. *According to* [23] *a valid* *p*-*value for testing* $H_{\ell }^{f}$, *under the assumption of positive regression dependency on subsets (PRDS) on the joint distribution of the* *m*_*ℓ*_ *individual* *p*-*values, can be defined as*

$$p^{u_{\ell }/m_{\ell }}=\min_{1\leq i\leq m_{\ell }-u_{\ell }+1}\left\{\frac{m_{\ell }-u_{\ell }+1}{i}p_{u_{\ell }-1+i:m_{\ell }}\right\}.$$ (3)

In general, a critical issue in connection with FDR control is the dependency structure among the *p*-values. The LSU test controls the FDR under the PRDS assumption regarding the joint distribution of the *p*-values, see [24] and [25]. It was shown in [26] that *φ*^*LSU*^ cannot be improved uniformly if the dependency among the *p*-values is completely unknown. Other procedures as the one introduced in [27] assume weak dependency in the sense of Definition 5.

**Definition 5 (Weak dependency)** *Let* *p*_1_, …, *p*_*m*_ *denote (random) marginal* *p*-*values for a collection* $H_{m}=\left\{H_{i},i\in I=\left\{1,\ldots ,m\right\}\right\}$ *of null hypotheses at hand. Let* *I*_*N*_ ⊆ *I* (*I*_*A*_ ⊆ *I*) with |*I*_*N*_| = *m*_*N*_ (|*I*_*A*_| = *m*_*A*_) *denote the index set of true (false) null hypotheses in* *I*. *Then*, *p*_1_, …, *p*_*m*_ *are called weakly dependent, if* *q*_*N*_ = lim_*m* → ∞_ *m*_*N*_/*m* *exists and*

$${\hat{F}}_{Nm_{N}}\left(t\right)=m_{N}^{-1}\sum_{i\in I_{N}}I_{\left[0,t\right]}\left(p_{i}\right)\to F_{N}\left(t\right),\;m\to \infty$$ (4)

$${\hat{F}}_{Am_{A}}\left(t\right)=m_{A}^{-1}\sum_{j\in I_{A}}I_{\left[0,t\right]}\left(p_{j}\right)\to F_{A}\left(t\right),\;m\to \infty ,$$ (5)

*where* $I_{S}$ *denotes the indicator function of the set* *S*, *convergence in* Eqs (4) and (5) *is uniform for* *t* ∈ [0, 1] *and almost sure, and* *F*_*N*_ *and* *F*_*A*_ *are continuous cumulative distribution functions with* 0 < *F*_*N*_(*t*) ≤ *t* *for all* *t* ∈ (0, 1]

Throughout this work, we assume that the *p*-values within each family are PRDS and weakly dependent. While one might argue against the weak dependency assumption in the fMRI context (cf. [28]), the validity of weak dependency for *p*-values corresponding to voxel data has been discussed in [29] on the basis of simulation studies for different magnitudes of positive correlation among the voxels. No situation militating against the assumption was found. The FDR behaviour of AORC-based multiple test procedures under the weak dependency assumption regarding the joint distribution of the *p*-values was investigated in Chapter 4 of [30].

### Considered two-stage multiple tests

In [16] a general method to design procedures coping with the selection of families has been provided. For a comparison with our proposed procedure *φ*^*HO*^ we make use of one of the so-called “simple selection adjusted procedures” proposed in [15], which is based on *φ*^*LSU*^ and is denoted throughout the remainder by *φ*^*Bog*^. Under suitable assumptions, this procedure achieves control of the FDR on the average over the selected families, FDR control within each family, and FDR control on the level of the families, see [15]. A simulation study in [31] suggests that global FDR control of *φ*^*Bog*^ holds in multi-phenotype genome-wide association studies which exhibit similar characteristics as the fMRI studies considered here.

**Algorithm 1 (The procedure *φ*^*Bog*^)**

1. *Test the* *k* *families with the LSU procedure at level* *α* *applied to* ${\left(p^{1/m_{\ell }}\right)}_{1\leq \ell \leq k}$, *see* Eq (3). *Obtain* *R* *rejections*.
2. *In the case of* *R* > 0, *apply in each of the* *R* *rejected families* *φ*^*LSU*^ *at level* *Rα*/*m*_*ℓ*_, *where ℓ denotes the index of a rejected family*.

We propose to apply the following procedure which harnesses the advantages of the AORC approach and exploits the structural information.

**Algorithm 2 (The procedure *φ*^*HO*^)** *Let* ⌊*x*⌋ *denote the largest integer smaller than or equal to* *x*.

1. *For a given tuning parameter* *κ* > *k*, let *u*_*ℓ*_ = ⌊*κ*^−1^ ⋅ *m*_*ℓ*_⌋ + 1 *for* 1 ≤ *ℓ* ≤ *k*. *Reject all families* $H_{\ell }$ *for which*
  $$p^{u_{\ell }/m_{\ell }}\leq \frac{\alpha }{\kappa }.$$
  *Obtain* *R* *rejections*.
2. *In the case of* *R* > 0, *apply in each of the* *R* *rejected families* $\varphi_{\lambda }^{AORC}$ *at level* *α*, *with* λ = *u*_*ℓ*_, *where ℓ denotes the index of a rejected family*.

Under standard assumptions which are typically made in FDR theory, all three aims of the statistical analyses (i. e., FDR control in each family $H_{\ell }$ separately, global FDR control, and FWER control on the group level) are achieved by *φ*^*HO*^, at least asymptotically as $\underset{1\leq \ell \leq k}{\text{min}}m_{\ell }\to \infty$; see S1 Appendix for details.

### Experiments

We will compare the two hierarchical procedures *φ*^*HO*^ and *φ*^*Bog*^ with AORC-based SUD tests regarding the empirical power on the combined set of hypotheses in Sections “Computer simulations regarding the power of *φ*^*HO*^” and “Power simulation”. In the simulations regarding fMRI data presented in Section “fMRI—Data” and “fMRI—Results”, we will make comparisons of *φ*^*LSU*^ with the hierarchical procedures on the combined set of voxels by means of their empirical FDRs. When evaluating real fMRI experiments, we compare the respective numbers of detections, i. e., rejections. The procedure *φ*^*LSU*^ and the AORC-based SUD tests will be applied to the combined set of voxels, neglecting the hierarchical structure.

#### Computer simulations regarding the power of *φ*^*HO*^

In this section we consider the performance of the procedures in terms of power of a multiple test. A standard notion of power of a multiple test procedure *φ*_(*m*)_ for *m* hypotheses is given in Definition 1.4 of [1] as

$${\text{power}}_{m}(\varphi_{\left(m\right)})=E\left[\frac{S_{m}}{m_{A}\vee 1}\right],$$

where *S*_*m*_ denotes the number of correct rejections and the expectation E refers to the true underlying measure. The global power of a multiple test procedure *φ*_(*m*)_ that operates on a structured family of hypotheses as considered in Section “Methods” is given by

$${\text{gpower}}_{m}\left(\varphi_{\left(m\right)}\right)=E\left[\frac{S_{m}}{m_{A}\vee 1}\right]=E\left[\frac{\sum_{\ell =1}^{k}S_{\ell }}{\sum_{\ell =1}^{k}m_{A\ell }\vee 1}\right],$$

where *m*_*Aℓ*_ and *S*_*ℓ*_ are the number of false null hypotheses and the number of correct rejections in family *ℓ*. For a given number *B* of Monte Carlo repetitions, the power of *φ*_(*m*)_ is estimated by the average value

$${\hat{\text{power}}}_{m}\left(\varphi_{\left(m\right)}\right)=\frac{1}{B}\sum_{b=1}^{B}\frac{s_{m,b}}{m_{A}},$$

where *s*_*m*, *b*_ denotes the realization of *S*_*m*_ in the *b*-th simulation run. In our simulations, we set *B* = 10,000 and *m* = 2,500.

The simulations refer to the one-sided normal means problem with $\Omega =R^{m}$, an observable random vector *T* = (*T*_1_, …, *T*_*m*_)^⊤^ with values in Ω such that $L\left(T\right)=N_{m}\left(\mu ,I_{m}\right)$, where *μ* = (*μ*_1_, …, *μ*_m_)^⊤^, and hypotheses

$$H_{j}:\left\{\mu_{j}=0\right\}\;\text{vs.}\;K_{j}:\left\{\mu_{j}>0\right\},j\in \left\{1,\cdots ,m\right\}.$$

The *p*-value for a hypothesis *H*_*j*_ is then given by

$$p_{j}\left(t_{j}\right)=P_{H_{j}}\left(T_{j}>t_{j}\right)=1-\Phi \left(t_{j}\right),$$

where *t*_*j*_ denotes the observed value of *T*_*j*_ and Φ denotes the cumulative distribution function of the standard normal distribution.

For convenience, we set all *μ*_*j*_, *j* ∈ *I*_*A*_, to the same value μ* > 0. The power of the different procedures will be investigated for different effect sizes μ*. The effect size μ* will range from 0.5 up to 5 in steps of 0.5. Furthermore, we assume that the family $H_{m}=\left(H_{1},\ldots ,H_{m}\right)$ is structured into two subfamilies $H_{m_{1}}$ and $H_{m_{2}}$. The parameter *κ* is set to 1,000, see Section “Power simulations” for justification. We let *π*_*ℓ*_ = *m*_*ℓ*_/*m* and *q*_*Nℓ*_ = *m*_*Nℓ*_/*m*_*ℓ*_, *ℓ* = 1, 2, where *m*_*Nℓ*_ denotes the number of true null hypotheses in family *ℓ*. Table 1 lists the considered parameter configurations. The FDR level was set to *α* = 5% in all simulations.

**Table 1:** Parameter configurations in the one-sided normal means problem.

	*π* = (*π*_1_, *π*_2_)	*q*_*N*_ = (*q*_*N*1_, *q*_*N*2_)
1	(0.5, 0.5)	(0.5, 0.5)
2	(0.5, 0.5)	(0.8, 0.1)
3	(0.8, 0.2)	(0.8, 0.1)
4	(0.5, 0.5)	(0.99, 0.01)
5	(0.8, 0.2)	(0.99, 0.01)

#### fMRI—Data

Simulations and analysis of experimental data were all performed within the **R** language and environment for statistical computing and graphics, cf. [32]. The **R**-scripts for the creation of the simulated data and their analysis are available from http://www.wias-berlin.de/preprint/2127/codeANDdata_2127.zip. *Simulated fMRI data*. We simulated fMRI data using the **R**-package **neuRosim** (cf. [33]) described in detail in [34]. The simulated data consisted of 105 volumes of size 20 × 20 × 20 isotropic voxels. The simulated stimulus had onset times at the 16-th, 46-th and 76-th volume, a duration overlapping 15 volumes and a repetition time of two seconds. The expected hemodynamic response to this block design was created using a convolution of the task indicator function with the standard “double-gamma” hemodynamic response function, see [35]. The “activation” region in this data was set to a sphere of radius 3 voxels. The center of the sphere was set in voxel coordinates (5, 5, 5) for Simulation A and in voxel (10, 10, 10) for Simulation B. Noise was added using a Rician distribution including spatial and temporal correlations.

We then analyzed the data with a standard general linear model (GLM) approach using the **R**-package **fmri** (cf. [36] and [37]) including corrections for temporal autocorrelations and quadratic signal trends. From the resulting statistical parametric map we determined local *p*-values.

We defined an arbitrary partition of the spatial domain into eight families of voxels corresponding to the eight “corners” of the data cube. For both simulation datasets we then applied the hierarchical testing procedures *φ*^*HO*^ and *φ*^*Bog*^, as well as *φ*^*LSU*^ at an FDR level of 0.05.

*Statistical Parametric Mapping (SPM) auditory fMRI test data*. For validation of our new inference method on experimental fMRI data we used a publicly available single subject fMRI dataset with an auditory stimulus design. The data can be downloaded at http://www.fil.ion.ucl.ac.uk/spm/data/auditory/ together with details on its acquisition.

The number of volumes at a repetition time of 7 seconds was 96 with alternating blocks of rest and auditory stimulus, starting with rest, each lasting for six volumes. Echo planar imaging (EPI) data was acquired on a modified 2T Siemens MAGNETOM Vision system. The spatial dimension of the data was 64 × 64 × 64 isotropic voxels of length 3mm. Calculation of local *p*-values was performed as described for the simulated fMRI data.

To define suitable families of voxels we normalized AFNI’s (cf. [38]) EPI template (TT_EPI-tlrc) in Talairach space with Brodmann labels to the functional data using the normalization toolbox of SPM8. Thus each voxel in the functional data was assigned a label according to the Brodmann atlas. Any other suitable atlas or definition of families could have been used here. We then applied the procedures *φ*^*HO*^, *φ*^*Bog*^, and *φ*^*LSU*^ to all voxels that had been assigned any label by the atlas matching described above, restricting analysis to the labelled cortex areas only.

*fMRI dataset using a sports imagination task*. We also re-used an fMRI dataset from [37] originating from an experiment performed with one healthy adult female subject. The data are publicly available under http://www.jstatsoft.org/v44/i11. The alternating design of rest and task blocks, starting with rest, was identical to the one of the simulated fMRI data and resulted in 105 volumes. The rest and task blocks had a duration of 30 seconds, the repetition time was 2 seconds. The task was imagination of playing tennis. The spatial dimension of the data cube was 64 × 64 × 30 with an in-plane resolution of 3.75mm and a slice thickness of 4mm. The echo time of the EPI sequence was 40ms and the flip angle was 80 degrees. Before the first rest block six dummy scans were discarded to allow for *T*_1_ saturation.

We repeated the analysis described for the SPM auditory fMRI test data, i.e., normalizing the Brodmann labels to the functional data using SPM8 and performing a standard GLM analysis with the **R**-package **fmri** to calculate local *p*-values. Signal detection was performed using the procedures *φ*^*HO*^, *φ*^*Bog*^, as well as *φ*^*LSU*^.

#### Other fMRI datasets

We also analyzed two more fMRI scans of another subject in a finger tapping task within the same task protocol as described for the sports imagination dataset. One of the datasets had a doubled in-plane resolution. The analysis yielded very similar results (with respect to the performance of the signal detection procedure) as the sports imagination dataset, which is why we decided not to show the results of the analysis here.

## Results

### Power simulations

**Fig 1:**
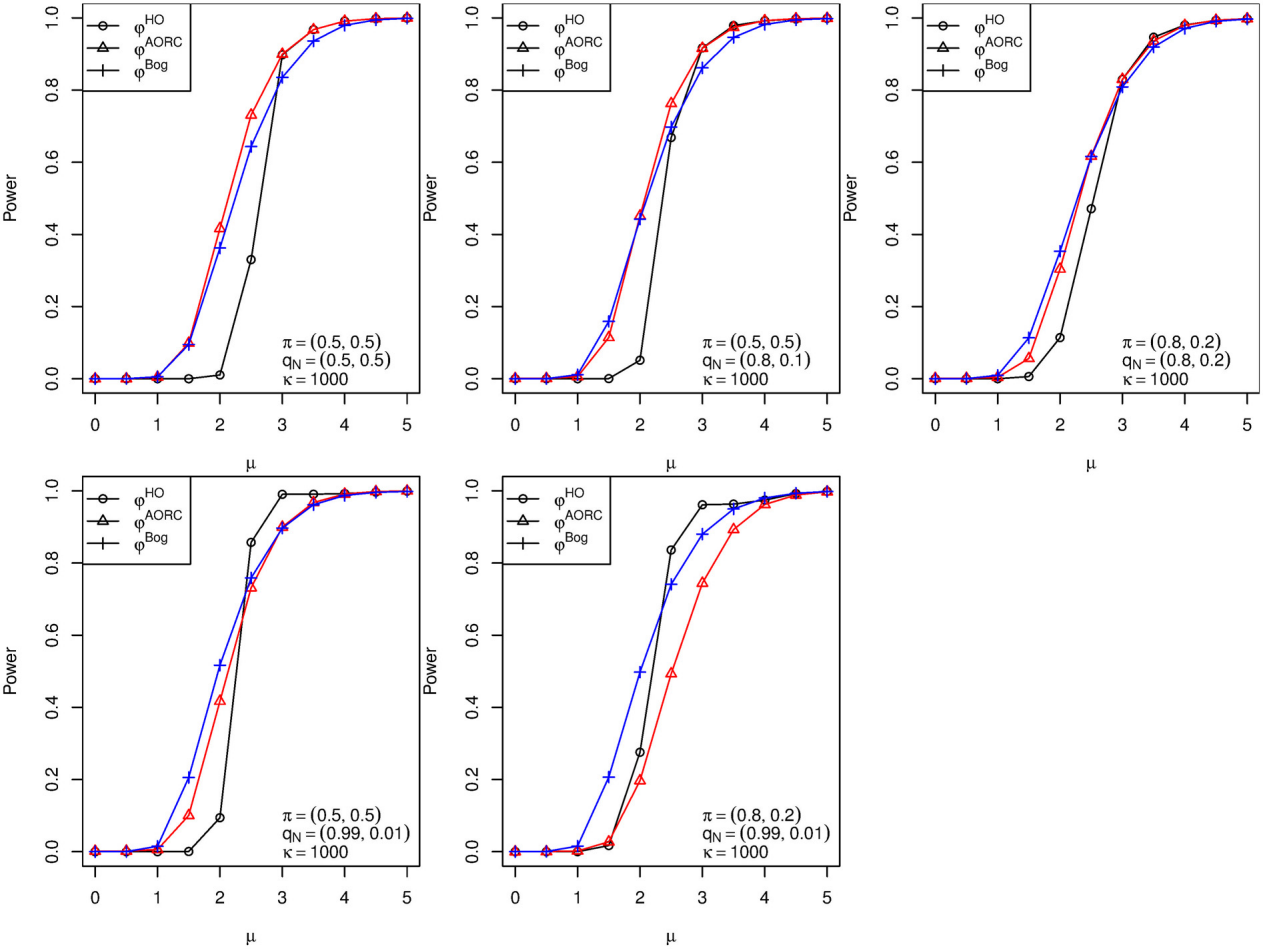
Empirical powers of the procedures. Empirical powers of the procedures *φ*^*HO*^ (black), *φ*^*Bog*^ (blue) and $\varphi_{u_{\ell }}^{AORC}$ (red) as a function of the effect size μ* in the one-sided normal means problem. The total number of hypotheses equals *m* = 2500, and the number of groups equals *k* = 2. The parameter configurations *π* = (*π*_1_, *π*_2_) and *q*_*N*_ = (*q*_*N*1_, *q*_*N*2_) are as in Table 1.

The five panels in Fig 1 refer to the five parameter configurations from Table 1 with the choice of *κ* = 1,000. This choice was made to ensure that the partial conjunction hypotheses coincide with the intersection hypotheses, for comparative purposes with the other procedures. For specific values of the proportion of true null hypotheses, the influence of *κ* on the performance of the procedure *φ*^*HO*^ is demonstrated in S1 Appendix.

In the second panel row of Fig 1 (comprising panels 4–5), the ratios *q*_*Nℓ*_, *ℓ* = 1, 2, are highly unbalanced. It can clearly be observed that this leads to an improvement in terms of power of the proposed procedure *φ*^*HO*^ over the existing multiple tests *φ*^*Bog*^ and $\varphi_{u_{\ell }}^{AORC}$, at least for μ* ∈ [2, 3]. In the first panel row (comprising panels 1–3), however, the empirical power of $\varphi_{u_{\ell }}^{AORC}$ is uniformly higher than that of *φ*^*Bog*^ and *φ*^*HO*^, respectively.

We may remark that a more detailed analysis of the decision patterns of the three concurring multiple tests (not shown here) revealed that the higher power of $\varphi_{u_{\ell }}^{AORC}$ in panels 2 and 3 is mainly due to the fact that *φ*^*Bog*^ and *φ*^*HO*^ discard the first family $H_{m_{1}}$ already in the first stage of the analysis (with high probability). Often, such a behavior is wanted in practice, because few isolated signals are typically interpreted as artifacts, especially in the fMRI context.

### fMRI—Results

#### fMRI—Simulations

**Fig 2:**
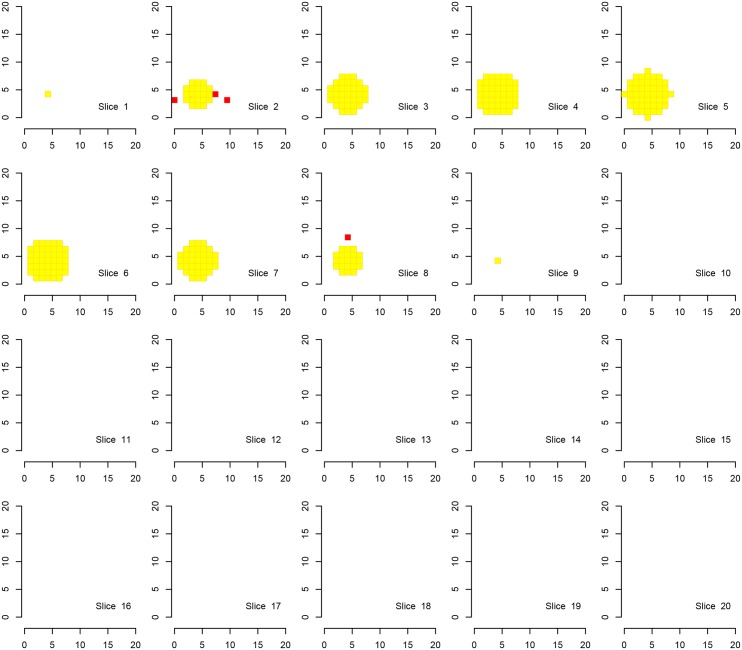
Discoveries of the procedure *φ*^*HO*^ in fMRI Simulation A. Discoveries of the procedure *φ*^*HO*^ in Simulation A on a cube with side length 20. There are eight disjoint families consisting of cubes with a side length of 10 voxels, each one located in one corner of the original cube. Shown are 20 slices corresponding to the third dimension. Ground activation (yellow) and the false rejections of *φ*^*HO*^ (red) are shown.

**Fig 3:**
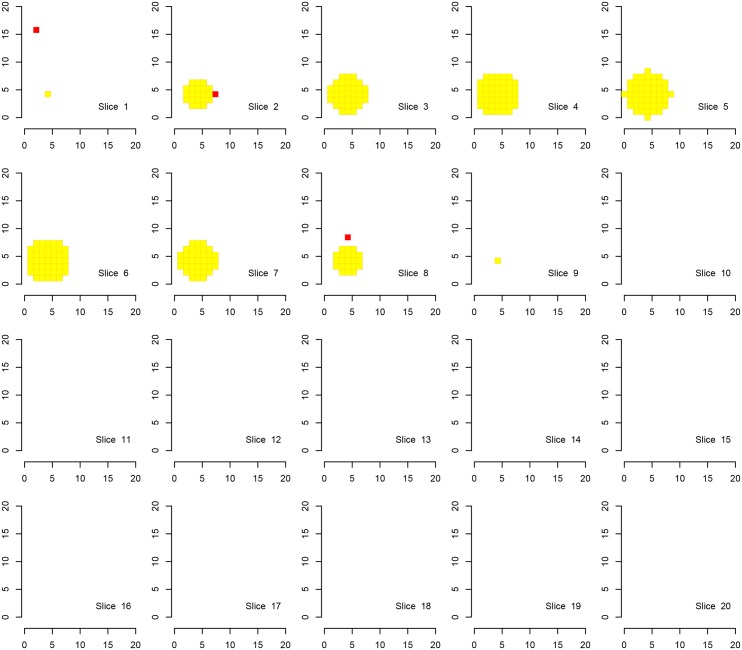
Discoveries of the procedure *φ*^*Bog*^ in fMRI Simulation A. Discoveries of the procedure *φ*^*Bog*^ in Simulation A on a cube with side length 20. There are eight disjoint families consisting of cubes with side length of 10 voxels, each one located in one corner of the original cube. Shown are 20 slices corresponding to the third dimension. Ground activation (yellow) and false rejections of *φ*^*Bog*^ (red) are shown.

**Fig 4:**
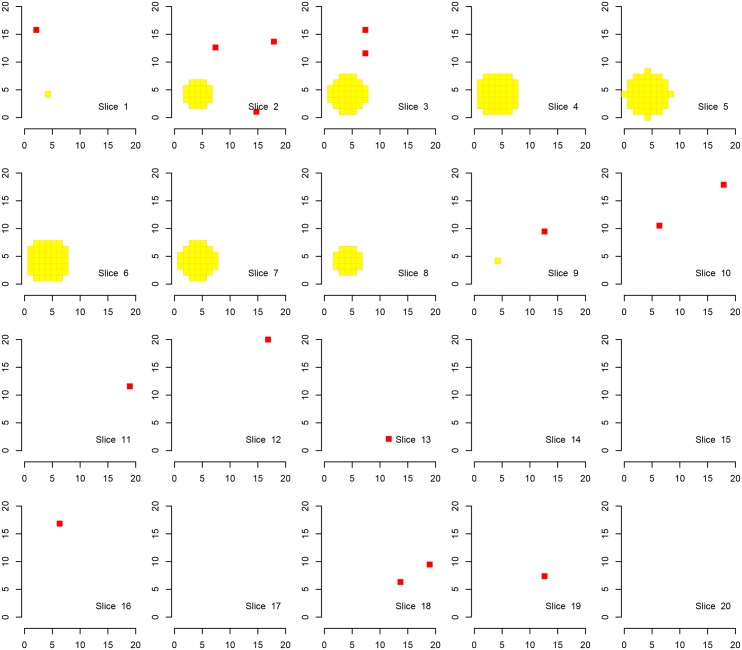
Discoveries of the procedure *φ*^*LSU*^ in fMRI Simulation A. Discoveries of the procedure *φ*^*LSU*^ in Simulation A on a cube with side length 20. There are eight disjoint families consisting of cubes with side length of 10 voxels, each one located in one corner of the original cube. Shown are 20 slices corresponding to the third dimension. Ground activation (yellow) and the false rejections of *φ*^*LSU*^ (red) are shown.

We first show the results for Simulation A, where the “activation area” is fully located within one of the defined families, in Figs 2, 3 and 4. Every procedure detects all true alternatives (which are marked in yellow), but we can observe a different number of false discoveries (indicated in red). The hierarchical procedure *φ*^*HO*^ does not make any discoveries in families without activation.

Comparing the detected activation areas with the known ground truth, we estimated the global and within-family false discovery rates as well as the mean FDR over the families for 1000 Monte Carlo repetitions. We can observe differences regarding the detection of false positives, see Table 2. The procedure *φ*^*LSU*^ has the most rejections, but violates the FDR in every family, except for the family in which the signal is located. All empirical FDRs are below 5% for the other two procedures.

**Table 2:** Global FDR, mean FDR and within family FDR in the fMRI Simulation A and B for the different procedures.

	Simulation A			Simulation B		
	*φ*^*HO*^	*φ*^*LSU*^	*φ*^*Bog*^	*φ*^*HO*^	*φ*^*LSU*^	*φ*^*Bog*^
gFDR	0.0352	0.0333	0.026	0.0362	0.0341	0.0347
FDR Fam. 1	0.0352	0.003	0.0257	0.0354	0.0191	0.0331
FDR Fam. 2	0	0.685	0.006	0.0357	0.0288	0.006
FDR Fam. 3	0	0.696	0.015	0.0356	0.0295	0.014
FDR Fam. 4	0	0.678	0.006	0.0331	0.0419	0.006
FDR Fam. 5	0.001	0.676	0.005	0.0354	0.0285	0.005
FDR Fam. 6	0	0.683	0.010	0.0363	0.0443	0.010
FDR Fam. 7	0	0.693	0.012	0.0355	0.0428	0.012
FDR Fam. 8	0	0.691	0.005	0.0337	0.0603	0.005
mean FDR	0.0045	0.6006	0.0106	0.0351	0.0369	0.0114

**Fig 5:**
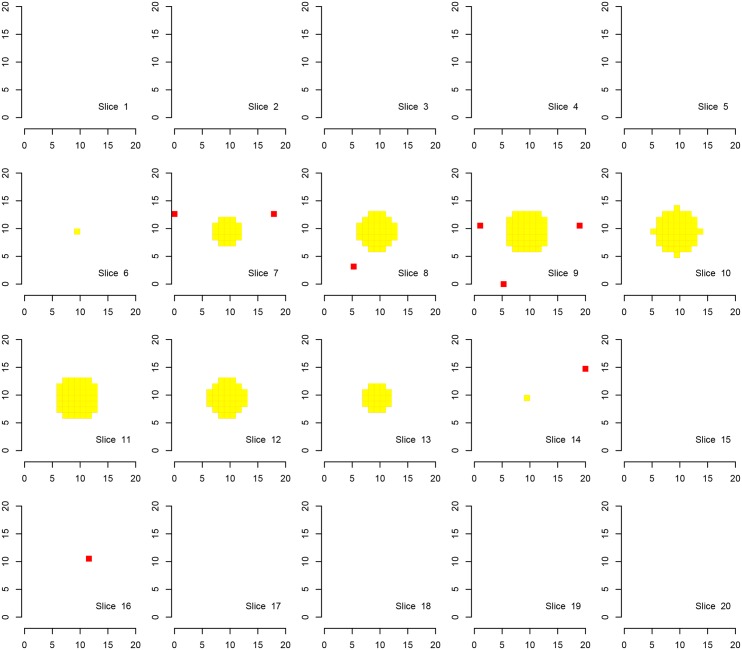
Discoveries of the procedure *φ*^*HO*^ in fMRI Simulation B. Discoveries of the procedure *φ*^*HO*^ in Simulation B on a cube with side length 20. There are eight disjoint families consisting of cubes with a side length of 10 voxels, each one located in one corner of the original cube. Ground activation (yellow) and the false rejections of *φ*^*HO*^ (red) are shown.

**Fig 6:**
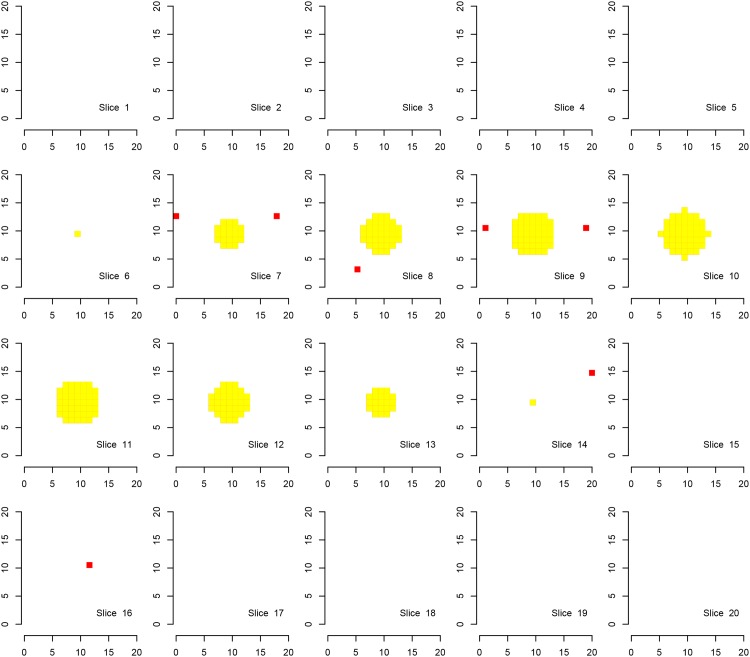
Discoveries of the procedure *φ*^*Bog*^ in fMRI Simulation B. Discoveries of the procedure *φ*^*Bog*^ in Simulation B on a cube with side length 20. There are eight disjoint families consisting of cubes with a side length of 10 voxels, each one located in one corner of the original cube. Ground activation (yellow) and the false rejections of *φ*^*Bog*^ (red) are shown.

**Fig 7:**
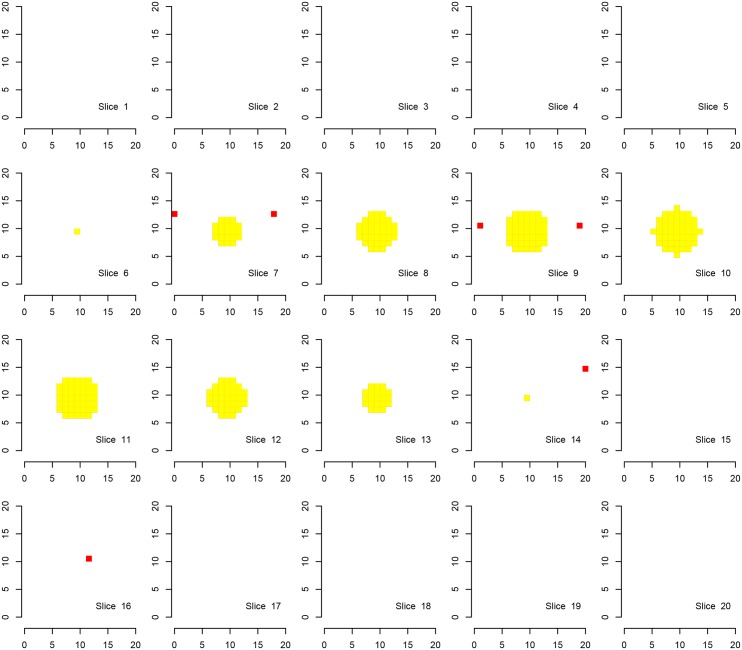
Discoveries of the procedure *φ*^*LSU*^ in fMRI Simulation B. Discoveries of the procedure *φ*^*LSU*^ in Simulation B on a cube with side length 20. There are eight disjoint families consisting of cubes with side length of 10 voxels, each one located in one corner of the original cube. Ground activation (yellow) and the false rejections of *φ*^*LSU*^ (red) are shown.

We show the detection results for Simulation B, where true activations are located within all defined families of voxels, in Figs 5, 6 and 7. In analogy to the presentations under Simulation A, we show the slices of activated voxels determined by the three different procedures overlayed with the true activation.

A visual inspection of the figures and the table confirms the desired behaviour of the procedure. In Table 2 we clearly observe that the families without activation (i. e., families 2–8 in Simulation A) are in most of the Monte Carlo repetitions excluded from the analysis by *φ*^*HO*^ and *φ*^*Bog*^. In contrast, activation is reported in all families when using the test *φ*^*LSU*^. It is not surprising that in families without signal the FDR in the family is not controlled for the LSU-procedure. If the signal is found in every family (Simulation B) there is no advantage in the use of the hierarchical approach. The order of magnitude regarding the FDRs seems to be the same for the two hierarchical procedures, although the attained FDR level of the procedure *φ*^*HO*^ is closer to 5%, suggesting higher power.

#### SPM auditory fMRI test data

**Fig 8:**
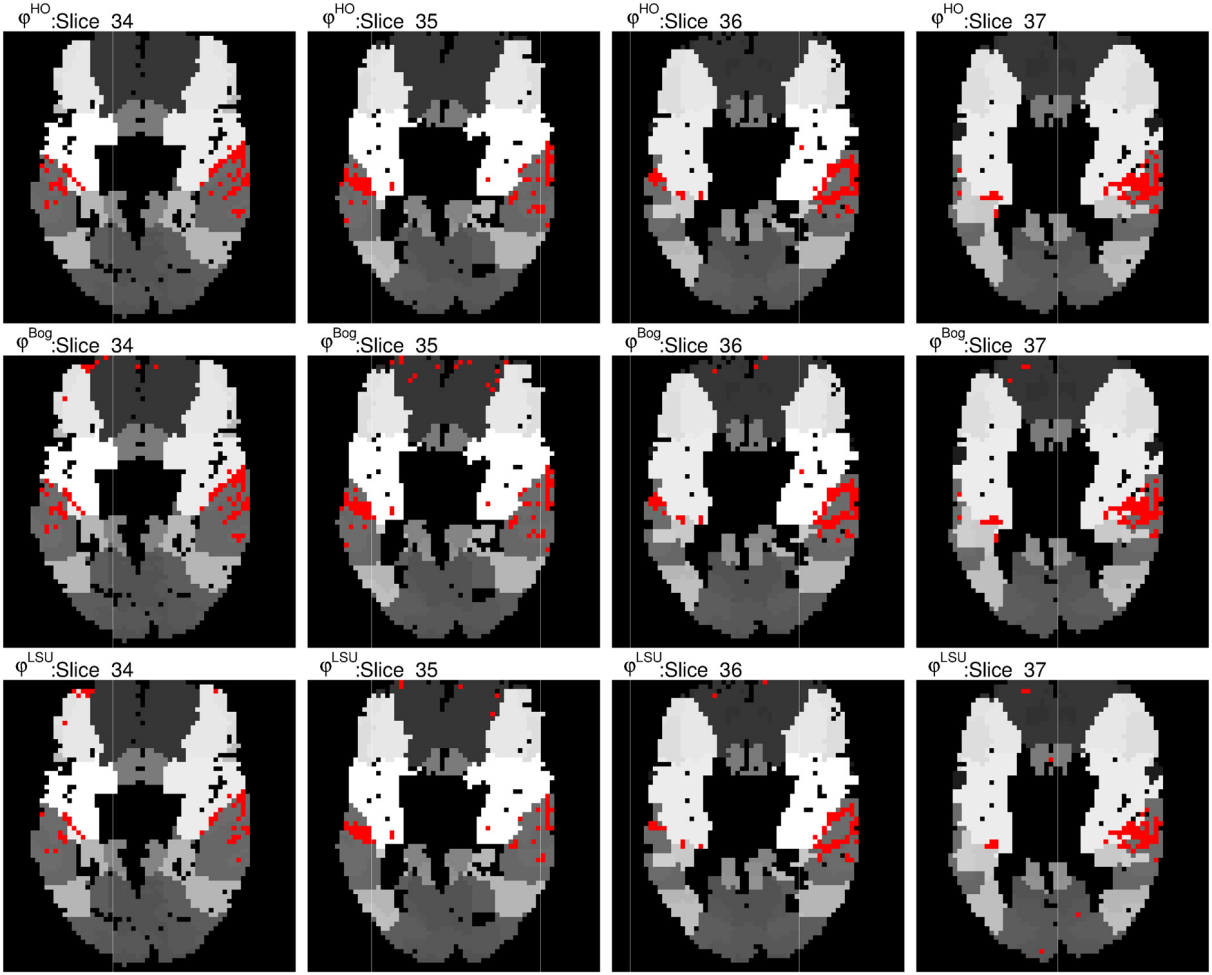
Discoveries on chosen slices of the brain for the SPM auditory fMRI dataset. We present chosen slices (auditory cortex visible) of the brain for the SPM auditory fMRI dataset and the discoveries proposed procedure *φ*^*HO*^ in the first row. The discoveries in the second row correspond to the procedure *φ*^*Bog*^ and in the third row the corresponding discoveries of *φ*^*LSU*^ are displayed.

We show the detection results in the auditory cortex of the proposed procedure *φ*^*HO*^ overlayed on the functional division of the brain according to the Brodmann atlas and compare them with the detections found by the procedures *φ*^*LSU*^ and *φ*^*Bog*^ in Fig 8. We can see that the hierarchical procedures detect voxels mainly located in the auditory areas, while the LSU procedure finds activations all over the brain. The full figures showing all slices can be found in S2, S3 and S4 Figs.

The table in S1 Table shows the number of discoveries in the different Brodmann areas. In agreement with Fig 8, it can be seen from this table that the proposed procedure leads to a far more concentrated signal detection in areas related to the auditory stimulus.

#### fMRI dataset using a sports imagination task

**Fig 9:**
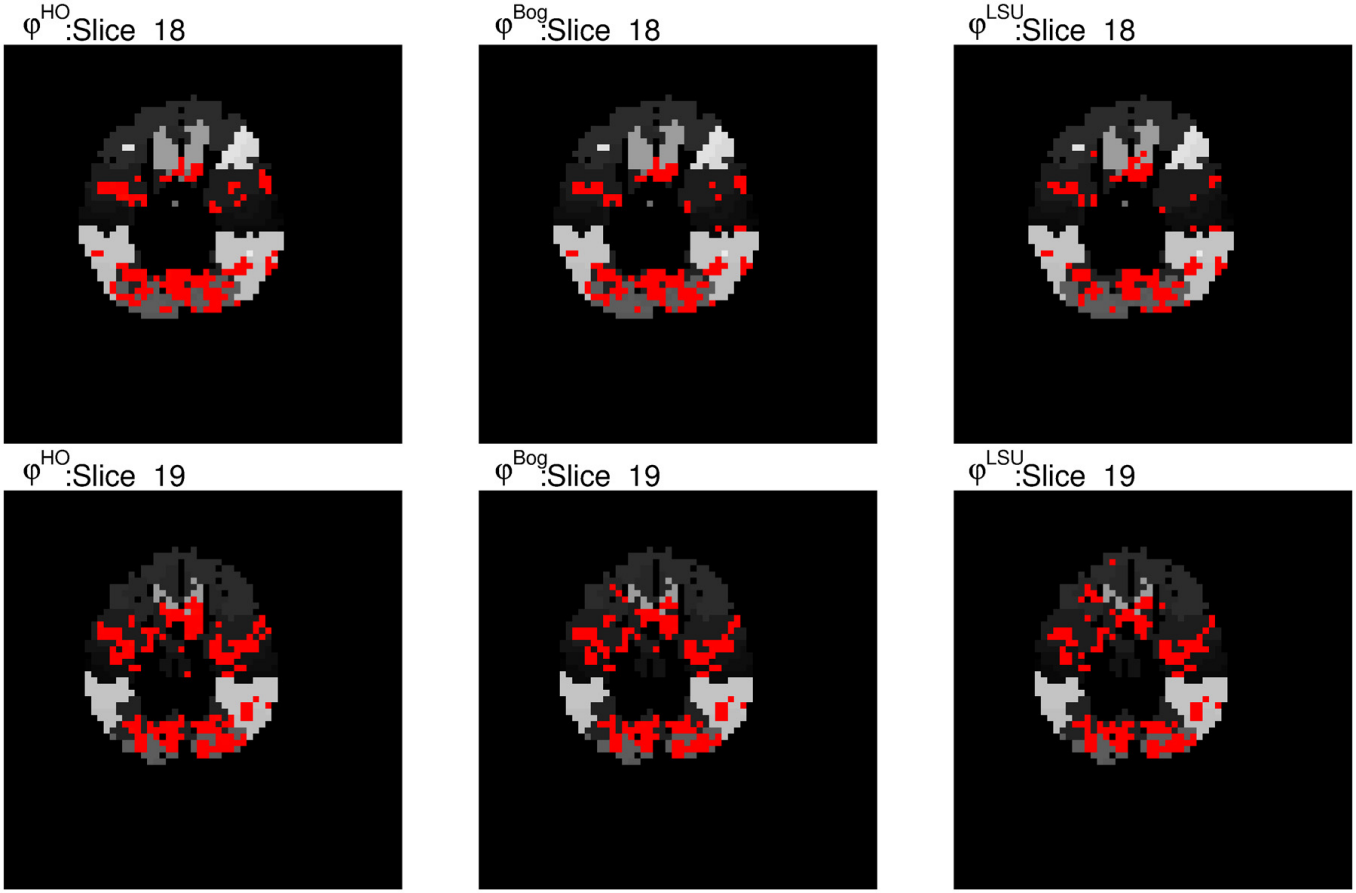
Discoveries on chosen slices of the brain for the sports imagination task dataset. We present chosen slices (motor cortex visible) of the brain for the sports imagination task and highlight the discoveries of the procedure *φ*^*HO*^ in the first column. The discoveries in the second column correspond to the procedure *φ*^*Bog*^ and in the third column the corresponding discoveries of *φ*^*LSU*^ are displayed.

We show the detection results of the proposed procedures overlayed on the Brodmann atlas. A visual inspection of Fig 9 reveals activation in the whole brain. As it can be seen in the table in S2 Table, in every area of the brain many activated voxels are detected by all procedures. We might hypothesize that the stimulus of this experiment, which is an imagination task, is related to much less specific activation due to its complexity. Similar to the situation in fMRI Simulation B we do not observe that the hierarchical procedures are more specific than *φ*^*LSU*^ regarding the Brodmann areas. The full figures are provided in S5, S6 and S7 Figs.

## Discussion

This work focused on the use of structural information in a new procedure to control the FDR. We provided a rigorous mathematical analysis of this new procedure and proved asymptotic control of the FDR. In simulations we studied the performance of the proposed method in situation with finite *m*. Furthermore, we applied it to simulated and real fMRI datasets.

For fMRI analysis our procedure bears the unique advantage of being specific to the families/regions in which brain activity is located and is highly sensitive within each family. This conclusion can be clearly drawn from Table 2 and is supported by the figures. Other FDR controlling procedures suffer from false positives in areas without signal. We first filter where strong signal can be found and continue to locate the voxels which are responsible for the strong signal, making use of the nonlinear critical values originating from the theory around the AORC. It was possible to demonstrate that when the activation is concentrated in a-priori known regions the procedure can be used to increase the specificity on the level of the families while finding a similar number of discoveries as the standard approaches within the families of interest. The hierarchical approach was demonstrated to perform close to the non-hierarchical approach if families do not differ in the number of true alternatives. However, we forfeit sensitivity for weak signals if the pre-test is not passed. The use of the Brodmann atlas for the real fMRI data is just a simple example of a division of the brain into functionally different regions, which can (and should) be replaced by more suitable selections in specific applications. In summary our procedure shows superior specificity during the detection of active regions of interest in the brain while being highly sensitive regarding the voxels within a detected region, suggesting good applicability of the FDR in signal detection in fMRI.

From a more general perspective, the proposed procedure *φ*^*HO*^ is designed to discard families which contain only few scattered signals. This may result in sub-optimal global power, but leads to higher specificity on the group level, compared with non-hierarchical procedures which test all *m* hypotheses together. Often, as in the fMRI context discussed above, the groups are the experimental units of interest, and in such a situation the hierarchical approach is recommendable. The test *φ*^*HO*^ depends on a tuning parameter *κ*, which has to be chosen by the researcher before the start of the analysis. A value *κ* ≤ *m*_*ℓ*_ for a family $H_{\ell }$ has the interpretation, that a family is declared active if there is evidence that it contains at least *m*_*ℓ*_/*κ* true alternatives. If *κ* > *m*_*ℓ*_ the partial conjunction hypothesis becomes the intersection hypothesis.

An interesting and challenging direction for future research is the consideration of additional layers of hierarchy in FDR-controlling multiple test procedures. For example, consider a hierarchical system $H_{m}$ of *m* hypotheses which is closed under intersection. In the case that FWER control at level *α* is targeted, the closure principle (see [39]) allows one to test all *m* hypotheses in $H_{m}$ at full level *α*, provided that the coherence rule is adhered to (rejection of a hypothesis $H_{i}\in H_{m}$ implies that all hypotheses in $H_{m}$ which are subsets of *H*_*i*_ are also rejected). How this principle can be transferred to the concept of (global) FDR control will be explored in future work.

## Supporting Information

S1 Appendix Mathematical derivations. Mathematical proofs and investigation of the proposed procedure regarding the tuning parameter *κ*.
(PDF)

S1 Table Discoveries in the SPM auditory experiment. Number of discoveries in the SPM auditory experiment overall and in each Brodmann area for the procedures *φ*^*HO*^, *φ*^*Bog*^, and *φ*^*LSU*^.
(XLS)

S2 Table Discoveries in the sports imagination task dataset. Number of discoveries in the sports imagination task dataset overall and in each Brodmann area for the procedures *φ*^*HO*^, *φ*^*Bog*^, and *φ*^*LSU*^.
(XLS)

S1 Fig Empirical power of *φ*^*HO*^. Empirical power of the procedure *φ*^*HO*^ for two different fractions *q*_*N*_ of true null hypotheses, as a function of the tuning parameter *κ* and the signal strength μ* in the normal means problem with variance 1.
(TIF)

S2 Fig Discoveries of *φ*^*HO*^ for the SPM auditory dataset. Discoveries of the proposed procedure *φ*^*HO*^ for the SPM auditory fMRI dataset on the Brodmann areas of the brain for all slices.
(TIF)

S3 Fig Discoveries of *φ*^*Bog*^ for the SPM auditory dataset. Discoveries of the procedure *φ*^*Bog*^ for the SPM auditory fMRI dataset on the Brodmann areas of the brain for all slices.
(TIF)

S4 Fig Discoveries of *φ*^*LSU*^ for the SPM auditory dataset. Discoveries of the procedure *φ*^*LSU*^ for the SPM auditory fMRI dataset on the Brodmann areas of the brain for all slices.
(TIF)

S5 Fig Discoveries of *φ*^*HO*^ for the sports imagination task dataset. Discoveries of the proposed procedure *φ*^*HO*^ for the sports imagination task dataset on the Brodmann areas of the brain for all slices.
(TIF)

S6 Fig Discoveries of *φ*^*Bog*^ for the sports imagination task dataset. Discoveries of the procedure *φ*^*Bog*^ for the sports imagination task dataset on the Brodmann areas of the brain for all slices.
(TIF)

S7 Fig Discoveries of *φ*^*LSU*^ for the sports imagination task dataset. Discoveries of the procedure *φ*^*LSU*^ for the sports imagination task dataset on the Brodmann areas of the brain for all slices.
(TIF)
