# Supplementary material for: More Specific Signal Detection in Functional Magnetic Resonance Imaging by False Discovery Rate Control for Hierarchically Structured Systems of Hypotheses
Source: PLoS One. 2016 Feb 25;11(2):e0149016. doi: 10.1371/journal.pone.0149016 (PMC4767609; doi:10.1371/journal.pone.0149016)
Supplement: S1 Appendix — Mathematical proofs and investigation of the proposed procedure regarding the tuning parameter κ. (PDF) [file pone.0149016.s001.pdf]

A Appendix

A.1 Mathematical proofs

First we introduce the basic setup and notation.

Model A.1. *Let $(\Omega, \mathcal{F}, \{\mathbb{P}_\vartheta : \vartheta \in \Theta\})$ be a statistical experiment and let $\mathcal{H} = \{H_1, \ldots, H_m\}$ denote a set of null hypotheses of interest with $\emptyset \neq H_i \subset \Theta$ for all $i \in \{1, \ldots, m\}$. Let $p_i, i \in \{1, \ldots, m\}$, denote the marginal p-value for testing H_i versus $K_i : \Theta \setminus H_i$. A (non-randomized) multiple test procedure $\varphi_{(m)} = (\varphi_1, \ldots, \varphi_m)^\top$ for testing $\mathcal{H}_m$ is a vector of measurable mappings (individual tests) from the sample space into $\{0, 1\}^m$. In this, the event $\{\varphi_i = 1\}$ means rejection of the i-th null hypothesis H_i. As convention, the index ℓ will be used to index families, while i is used to index individual hypotheses.*

Relevant quantities.

Definition A.1. *Under the assumptions of Model A.1, we let the total number of rejections, the number of erroneous rejections, the number of correct rejections, and the* FDR*, respectively, of $\varphi_{(m)}$ be defined as*

$$R_m(\varphi_{(m)}) = |\{i \in \{1, \ldots, m\} : \varphi_i = 1\}|, \quad \text{(A.1)}$$

$$V_m(\varphi_{(m)}) = |\{i \in \{1, \ldots, m\} : \varphi_i = 1 \text{ and } H_i \text{ is true}\}|, \quad \text{(A.2)}$$

$$S_m(\varphi_{(m)}) = |\{i \in \{1, \ldots, m\} : \varphi_i = 1 \text{ and } H_i \text{ is false}\}|, \quad \text{(A.3)}$$

$$\text{FDR}_\vartheta(\varphi_{(m)}) = \mathbb{E}_\vartheta \left[\frac{V_m(\varphi_{(m)})}{R_m(\varphi_{(m)}) \vee 1} \right]. \quad \text{(A.4)}$$

The multiple test $\varphi_{(m)}$ is said to control the FDR *at level $\alpha \in (0, 1)$ if*

$$\sup_{\vartheta \in \Theta} \text{FDR}_\vartheta(\varphi_{(m)}) \leq \alpha.$$

It is said to control the FDR *asymptotically at level α as $m \to \infty$ if*

$$\limsup_{m \to \infty} \sup_{\vartheta \in \Theta} \text{FDR}_\vartheta(\varphi_{(m)}) \leq \alpha.$$

If the m hypotheses are structured in disjoint families $\mathcal{H}_1, \ldots, \mathcal{H}_k$ with $|\mathcal{H}_\ell| = m_\ell$ for $1 \leq k \leq m$, a multiple test $\varphi_{(m_\ell)}$ is applied within each family, and we set $\varphi_{(m)} = (\varphi_{(m_1)}, \ldots, \varphi_{(m_k)})^\top$, we define the global FDR *of $\varphi_{(m)}$ by*

$$\text{gFDR}_\vartheta(\varphi_{(m)}) = \mathbb{E}_\vartheta \left[\frac{\sum_{\ell=1}^k V_{m_\ell}(\varphi_{(m_\ell)})}{\left\{ \sum_{\ell=1}^k R_{m_\ell}(\varphi_{(m_\ell)}) \right\} \vee 1} \right].$$

In the sequel, all considered multiple test procedures are such that the quantities in (A.1) - (A.4) actually only depend on the joint distribution of the (random) p-values $p_1, \ldots, p_m$, and one may assume that $(\Omega, \mathcal{F}) = ([0, 1]^m, \mathcal{B}([0, 1]^m))$ without loss of generality.

Critical value functions and rejection curves. The critical values $\alpha_{i:m}$ from Definition 2 may be defined in terms of a critical value function $\rho : [0,1] \to [0,1]$, where ρ is non-decreasing and continuous, $\rho(0) = 0$ and $\alpha_{i:m} = \rho(i/m), i \in \{1,\dots,m\}$. For a given critical value function ρ, the function r defined by $r(t) = \inf\{u : \rho(u) = t\}$ for $t \in [0,1]$ is called the rejection curve corresponding to ρ.

The AORC $r_\alpha : [0,1] \to [0,1]$ is defined by

$$r_\alpha(t) = \frac{t}{t(1-\alpha)+\alpha}, \quad t \in [0,1],$$

and the corresponding critical value function is given by $r_\alpha^{-1}(t) = 1 - r_\alpha(1-t)$, see Finner et al. [2009]. The critical values induced by this critical value function are the ones given in Definition 3.

Lemma A.1 (Sen [1999]). *Denote the empirical cumulative distribution function (ecdf) of the p-values $p_1,\dots,p_m$ by $\hat{F}_m$, given by*

$$\hat{F}_m(t) = \sum_{i=1}^{m} \mathbb{I}_{[0,t]}(p_i).$$

Assume that $\alpha_{i:m} = \rho(i/m), i \in \{1,\dots,m\}$ for a critical value function ρ with corresponding rejection curve r. Then it holds

$$p_{i:m} \leq \alpha_{i:m} \text{ if and only if } \hat{F}_m(p_{i:m}) \geq r(p_{i:m}).$$

Additional technical assumptions. Let $m_{N\ell}$ denote the number and $q_{N\ell}(m_\ell) = m_{N\ell}/m_\ell$ the proportion of true null hypotheses in family $\ell \in \{1,\dots,k\}$. Define $\pi_\ell(m) = m_\ell/m$ as the proportion of hypotheses belonging to family ℓ. Consider an asymptotic setting such that $\forall \ell \in \{1,\dots,k\}$: $m_\ell \to \infty$. For convenience, we assume $\pi_\ell(m) \to \pi_\ell \in (0,1)$ and $q_{N\ell}(m_\ell) \to q_{N\ell} \in [0,1]$.

Let $\vartheta^* = \vartheta^*(m_{N1},\dots,m_{Nk})$ denote a parameter value such that for every family $\mathcal{H}_\ell$, $1 \leq \ell \leq k$, the $m_{N\ell}$ p-values corresponding to true null hypotheses are uniformly distributed on $[0,1]$ and jointly stochastically independent, and that the remaining $(m_\ell - m_{N\ell})$ p-values corresponding to false null hypotheses are almost surely equal to zero. Such a parameter value is commonly referred to as a Dirac-uniform configuration, see, e. g., Section 2.2.2 of Dickhaus [2014] and references therein. Notice that ϑ^* does not necessarily have to be contained in Θ. Under ϑ^*, the ecdf of the m_ℓ p-values in family $\mathcal{H}_\ell$, say $\hat{F}_{m_\ell,\ell}$, converges in the Glivenko-Cantelli sense to $\hat{F}_{\infty,\ell}$, given by $\hat{F}_{\infty,\ell}(t) = (1-q_{N\ell}) + q_{N\ell}t$, $t \in [0,1]$. Furthermore, r_α and $\hat{F}_{\infty,\ell}$ possess a unique point of intersection on $[0,1)$, cf. Figure 5.2 of Dickhaus [2014]. We denote by $t_{q_{N\ell}}$ the abscissa of this point of intersection. In general $t = \alpha_{i:m}$ is called a crossing point between $\hat{F}_m$ and r if it satisfies $\hat{F}_m(p_{i:m}) \geq r(p_{i:m})$ and $\hat{F}_m(p_{i+1:m}) < r(p_{i+1:m})$ for $i \in \{1,\dots,m-1\}$ or $\hat{F}_m(p_{m:m}) \geq r(p_{m:m})$ for $i = m$.

Finally, we introduce the following assumption regarding the type I error behavior of φ^{HO} with respect to the parameter ϑ of the statistical model.

Assumption A.1. *For given numbers $m_{N1},\dots,m_{Nk}$, the parameter value $\vartheta^* = \vartheta^*(m_{N1},\dots,m_{Nk})$ is a least favorable parameter configuration (LFC) for the FDR of $\varphi^{HO}_{(m_\ell)}$, $1 \leq \ell \leq k$, at least asymptotically as $\min_{1\leq\ell\leq k} m_\ell \to \infty$, where $\varphi^{HO}_{(m_\ell)}$ denotes the proposed two-stage test applied in family $\mathcal{H}_\ell$. This means that $\text{FDR}_\vartheta(\varphi^{HO}_{(m_\ell)}) \leq \text{FDR}_{\vartheta^*}(\varphi^{HO}_{(m_\ell)})$ for all ϑ which are such that exactly $m_{N\ell}$ null hypotheses are true in family $\mathcal{H}_\ell$, $1 \leq \ell \leq k$.*

Assumption A.1 is a standard assumption in FDR theory; see, among others, Blanchard et al. [2014] and Bodnar and Dickhaus [2014] and references therein.

Main results.

Theorem A.1. *Let $\vartheta \in \Theta$ and assume that for $1 \leq \ell \leq k$ the multiple test $\varphi_{(m_\ell)}$ is an SUD test based on the critical value function $\rho \leq r_\alpha^{-1}$ (with corresponding rejection curve r). Furthermore, let the assumptions from above be fulfilled and let $\varphi_{(m)} = (\varphi_{(m_1)}, \ldots, \varphi_{(m_k)})^\top$. For notational convenience, let $R_{m_\ell} = R_{m_\ell}(\varphi_{(m_\ell)})$ and $V_{m_\ell} = V_{m_\ell}(\varphi_{(m_\ell)})$.*

If

$$\forall \ell \in \{1, \ldots, k\} : \lim_{m_\ell \to \infty} \mathbb{P}_\vartheta \left(\frac{R_{m_\ell}}{m_\ell} \in (0, r_\alpha(t_{q_{N\ell}(m_\ell)})] \right) = 1,$$

then it holds that

$$\limsup_{m \to \infty} \text{gFDR}_\vartheta(\varphi_{(m)}) \leq \alpha.$$

Proof. The global FDR computes as

$$\text{gFDR}_\vartheta(\varphi_{(m)}) = \mathbb{E}_\vartheta \left[\frac{\sum_{\ell=1}^k V_{m_\ell}}{\left\{ \sum_{\ell=1}^k R_{m_\ell} \right\} \vee 1} \right] = \mathbb{E}_\vartheta \left[\frac{m^{-1} \sum_{\ell=1}^k V_{m_\ell}}{m^{-1} \left(\left\{ \sum_{\ell=1}^k R_{m_\ell} \right\} \vee 1 \right)} \right]. \tag{A.5}$$

Let $t_{m_\ell} \in [0, 1]$ denote the random crossing point between r and the ecdf of the p-values $\hat{F}_{m_\ell, \ell}$ characterizing the rejection rule of $\varphi_{(m)}$. This allows for the representation $R_{m_\ell}/m_\ell = r(t_{m_\ell}) = \hat{F}_{m_\ell, \ell}(t_{m_\ell})$ and $V_{m_\ell} = m_{N\ell} \hat{F}_{Nm_\ell, \ell}(t_{m_\ell})$. This means that the right-hand side of (A.5) equals

$$\mathbb{E}_\vartheta \left[\frac{\sum_{\ell=1}^k \pi_\ell(m) q_{N\ell} \hat{F}_{Nm_\ell, \ell}(t_{m_\ell})}{\sum_{\ell=1}^k \pi_\ell(m) r(t_{m_\ell})} \right] = \mathbb{E}_\vartheta \left[\frac{\sum_{\ell=1}^k \pi_\ell(m) q_{N\ell} \hat{F}_{Nm_\ell, \ell}(t_{m_\ell}) r(t_{m_\ell}) / r(t_{m_\ell})}{\sum_{\ell=1}^k \pi_\ell(m) r(t_{m_\ell})} \right]. \tag{A.6}$$

An argumentation analogous to the one in the proof of Theorem 4.5 in Gontscharuk [2010] allows us to find an asymptotic non random upper bound for $q_{N\ell} \hat{F}_{Nm_\ell}(t_{m_\ell}) / r(t_{m_\ell})$. According to (5) in Definition 5, we can choose a $\delta > 0$ and m_ℓ large enough such that $\sup_{t \in [0,1]} |\hat{F}_{Nm_\ell}(t) - F_N(t)| \leq \delta$. Then it holds that

$$q_{N\ell} \hat{F}_{Nm_\ell}(t_{m_\ell}) / r(t_{m_\ell}) \leq q_{N\ell} t_{m_\ell} / r(t_{m_\ell}) + \mathcal{O}(\delta) \leq q_{N\ell} t_{q_{N\ell}} / r_\alpha(t_{q_{N\ell}}) + \mathcal{O}(\delta).$$

By design of the function r_α, it holds that $q_{N\ell} t_{q_{N\ell}} / r_\alpha(t_{q_{N\ell}}) = \min\{\alpha, q_{N\ell}\}$. Thus, it holds that the right-hand side of (A.6) can for eventually all large m_ℓ be bounded from above by

$$\mathbb{E}_\vartheta \left[\frac{\sum_{\ell=1}^k \pi_\ell(m) r_\alpha(t_{m_\ell}) \min\{\alpha, q_{N\ell}\}}{\sum_{\ell=1}^k \pi_\ell(m) r_\alpha(t_{m_\ell})} \right] + \mathcal{O}(\delta).$$

Since δ can be chosen arbitrarily small, this entails

$$\limsup_{m \to \infty} \text{gFDR}_\vartheta(\varphi_{(m)}) \leq \alpha.$$

■

Theorem A.2 (Statistical properties of the procedure φ^{HO}). *Assume that the assumptions from above are fulfilled. Then, the proposed procedure φ^{HO} defined by Algorithm 2 controls the* FWER *at the stage of the families at level α. Furthermore, the global* FDR *of φ^{HO} and the* FDR *of φ^{HO} within each family are asymptotically bounded by α.*

Proof. Recall that the family $\mathcal{H}_\ell$ is selected at the first stage of analysis if and only if the corresponding conjunction p-value p^{u_ℓ/m_ℓ} does not exceed α/κ. Since $\kappa > k$, the Bonferroni inequality yields the first assertion.

In order to show asymptotic control of the global FDR, assume first that $q_{N\ell} < 1$ for all $1 \leq \ell \leq k$. We notice that every hypothesis which is rejected by $\varphi^{HO}_{(m_\ell)}$ would also be rejected by $\varphi^{AORC}_{u_\ell,(m_\ell)}$ alone, where $\varphi^{AORC}_{u_\ell,(m_\ell)}$ denotes the SUD test which is applied in family $\mathcal{H}_\ell$ in the second stage of $\varphi^{HO}_{(m_\ell)}$, $1 \leq \ell \leq k$. This follows from the fact that κ and hence, u_ℓ, are fixed constants and the rejection rule of $\varphi^{HO}_{(m_\ell)}$ involves the additional condition regarding p^{u_ℓ/m_ℓ}. Hence, $R_{m_\ell}(\varphi^{HO}_{(m_\ell)}) \leq R_{m_\ell}(\varphi^{AORC}_{u_\ell,(m_\ell)})$. Under ϑ^* (cf. Assumption A.1) and by construction of r_α, we have, by setting $t_{q_{N\ell}} = 1$ for $q_{N\ell} < \alpha$, that $R_{m_\ell}(\varphi^{AORC}_{u_\ell,(m_\ell)})/m_\ell \to r_\alpha(t_{q_{N\ell}})$ almost surely, cf. Corollary 5.1.(i) of Finner et al. [2009]. We conclude that $\limsup_{m_\ell\to\infty} R_{m_\ell}(\varphi^{HO}_{(m_\ell)})/m_\ell \leq r_\alpha(t_{q_{N\ell}})$ for all $\vartheta \in \Theta$. On the other hand, consider for each $1 \leq \ell \leq k$ such that $\mathcal{H}_\ell$ has been selected at the first stage of analysis the following chain of inequalities:

$$\begin{aligned} p_{u_\ell:m_\ell} &\leq \min_{j=1,\ldots,(m_\ell-u_\ell+1)} \left\{ p_{(u_\ell-1+j):m_\ell} \right\} \\ &\leq p^{u_\ell/m_\ell} = \min_{j=1,\ldots,(m_\ell-u_\ell+1)} \left\{ \frac{(m_\ell - u_\ell + 1)}{j} p_{(u_\ell-1+j):m_\ell} \right\} \\ &\leq \frac{\alpha}{\kappa} \leq r_\alpha^{-1}\left(\frac{m_\ell/\kappa}{m_\ell}\right) \leq r_\alpha^{-1}\left(\frac{\lfloor 1/\kappa \cdot m_\ell \rfloor + 1}{m_\ell}\right) = r_\alpha^{-1}\left(\frac{u_\ell}{m_\ell}\right). \end{aligned}$$

Thus, if the family $\mathcal{H}_\ell$ is rejected, the SUD procedure $\varphi^{AORC}_{u_\ell,(m_\ell)}$ will reject at least u_ℓ hypotheses within $\mathcal{H}_\ell$. Notice that, by definition of u_ℓ, we have that $u_\ell/m_\ell \geq \kappa^{-1}$. We conclude that, in each selected family $\mathcal{H}_\ell$, $\liminf_{m_\ell\to\infty} R_{m_\ell}(\varphi^{HO}_{(m_\ell)})/m_\ell > 0$. Thus, Theorem A.1 can be applied with k replaced by $|\{1 \leq \ell \leq k : \mathcal{H}_\ell \text{ has been rejected}\}|$ in this case.

However, if for some $\ell \in \{1, \ldots, k\}$ we have $q_{N\ell} = 1$, we can find a number m_ℓ which is large enough such that $\mathbb{P}_\vartheta(p^{u_\ell/m_\ell} \leq \alpha/\kappa) \leq \alpha/\kappa$ due to the assumed validity of the conjunction p-value. Hence, letting x denote all observed data, straightforward calculation yields for ϑ, which is such that $q_{N\ell} = 1$, that

$$\begin{aligned} \mathbb{E}_\vartheta\left[\frac{V_{m_\ell}}{R_{m_\ell} \vee 1}\right] &= \int \frac{V_{m_\ell}(x)}{R_{m_\ell}(x) \vee 1} d\mathbb{P}_\vartheta(x) \\ &= \int_{\{p^{u_\ell/m_\ell} \leq \alpha/\kappa\}} \frac{V_{m_\ell}(x)}{R_{m_\ell}(x) \vee 1} d\mathbb{P}_\vartheta(x) + \int_{\{p^{u_\ell/m_\ell} > \alpha/\kappa\}} \frac{V_{m_\ell}(x)}{R_{m_\ell}(x) \vee 1} d\mathbb{P}_\vartheta(x) \\ &\leq \int_{\{p^{u_\ell/m_\ell} \leq \alpha/\kappa\}} 1 d\mathbb{P}_\vartheta + 0 \\ &= \mathbb{P}_\vartheta(p^{u_\ell/m_\ell} \leq \alpha/\kappa) \leq \alpha/\kappa, \end{aligned}$$

which completes the argumentation.

Asymptotic FDR control within each family can be established as follows. If a family $\mathcal{H}_\ell$ is not rejected, we have $R_{m_\ell}(\varphi^{HO}_{(m_\ell)}) = V_{m_\ell}(\varphi^{HO}_{(m_\ell)}) = 0$. On the other hand, in each selected family $\mathcal{H}_\ell$, it holds $V_{m_\ell}(\varphi^{HO}_{(m_\ell)}) \leq V_{m_\ell}(\varphi^{AORC}_{u_\ell,(m_\ell)})$ by the same argumentation as for $R_{m_\ell}(\varphi^{HO}_{(m_\ell)})$. Under the LFC ϑ^*, this also entails that

$$\frac{V_{m_\ell}(\varphi^{HO}_{(m_\ell)})}{R_{m_\ell}(\varphi^{HO}_{(m_\ell)}) \vee 1} \leq \frac{V_{m_\ell}(\varphi^{AORC}_{u_\ell,(m_\ell)})}{R_{m_\ell}(\varphi^{AORC}_{u_\ell,(m_\ell)}) \vee 1}$$

almost surely, because the structure of an SUD test yields that, as soon as $V_{m_\ell}(\varphi^{HO}_{(m_\ell)}) \geq 1$, we have $R_{m_\ell}(\varphi^{HO}_{(m_\ell)}) = V_{m_\ell}(\varphi^{HO}_{(m_\ell)}) + (m_\ell - m_{N\ell})$, and the mapping $x \mapsto x/(x+a)$ is isotone in $x > 0$ for $a \geq 0$. Since $\varphi^{AORC}_{u_\ell,(m_\ell)}$ asymptotically controls the FDR under ϑ^*, this implies the assertion. ■

A.2 The tuning parameter κ

Here, we report results of a power study regarding the tuning parameter κ. The study was done in two setups for the normal means problem with effect size μ^* and variance 1, analogous to the simulations in "Computer simulations regarding the power of φ^{HO}". Our theoretical investigations indicate that we can expect the power of the procedure φ^{HO} within one selected family $\mathcal{H}_\ell$ (in our case of size $m_\ell = 2{,}000$) to depend on the ratio of true null hypotheses $q_{N\ell}$ within the family. To this end, we considered a balanced and a highly unbalanced case by setting $q_{N\ell} \in \{0.5, 0.99\}$. In both cases the power of φ^{HO} has been estimated as a function of $\mu^* \in [0,5]$, and we let the parameter κ range from 1 to $10{,}000{,}000$ on a $\log_{10}$ scale.

The plots in S1 Fig. indicate that small values of κ lead to a high specificity in case of a large value of $q_{N\ell}$, while large values of κ lead to a good sensitivity in case of a moderate value of $q_{N\ell}$. This is line with the recommendation that κ should be chosen according to the amount of signals within a family which is considered relevant.

References

Blanchard, G., Dickhaus, T., Roquain, E., Villers, F., 2014. On least favorable configurations for step-up-down tests. Stat. Sin. 24 (1), 1–23.

Bodnar, T., Dickhaus, T., 2014. False discovery rate control under Archimedean copula. Electron. J. Stat. 8 (2), 2207–2241.

Dickhaus, T., 2014. Simultaneous statistical inference. With applications in the life sciences. Berlin: Springer.

Finner, H., Dickhaus, T., Roters, M., 2009. On the false discovery rate and an asymptotically optimal rejection curve. Ann. Stat. 37 (2), 596–618.

Gontscharuk, V., 2010. Asymptotic and Exact Results on FWER and FDR in Multiple Hypotheses Testing. Ph.D. thesis, Heinrich-Heine-Universität Düsseldorf.

Sen, P., 1999. Some remarks on simes-type multiple test of significance. J. Stat. Plan. Inference 82, 139 –145.
