# Supplementary figures and images for: More Specific Signal Detection in Functional Magnetic Resonance Imaging by False Discovery Rate Control for Hierarchically Structured Systems of Hypotheses
Source: PLoS One. 2016 Feb 25;11(2):e0149016. doi: 10.1371/journal.pone.0149016 (PMC4767609; doi:10.1371/journal.pone.0149016)

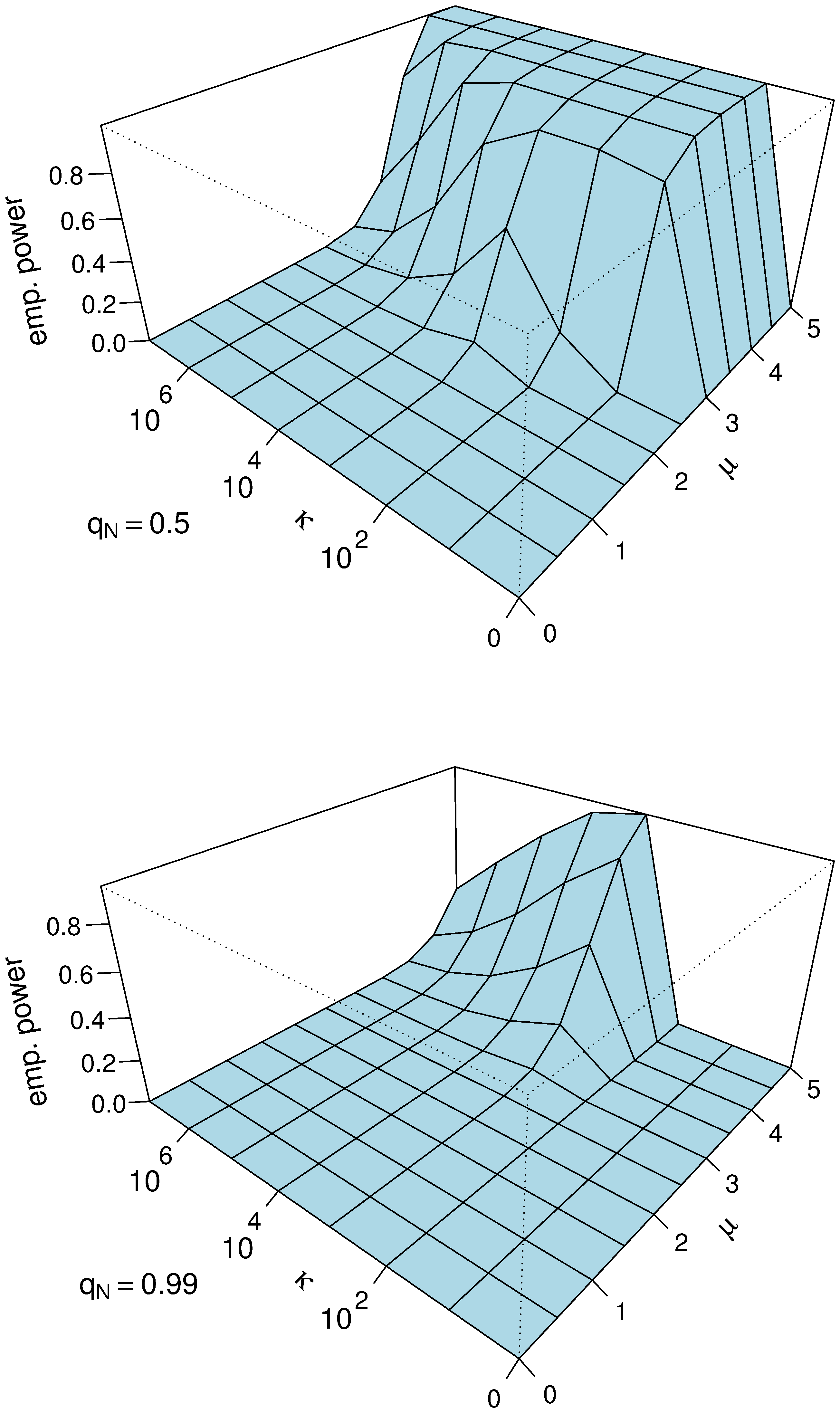

Supplement: S1 Fig — Empirical power of the procedure φHO for two different fractions qN of true null hypotheses, as a function of the tuning parameter κ and the signal strength μ* in the normal means problem with variance 1. (TIF) [file pone.0149016.s004.tif]

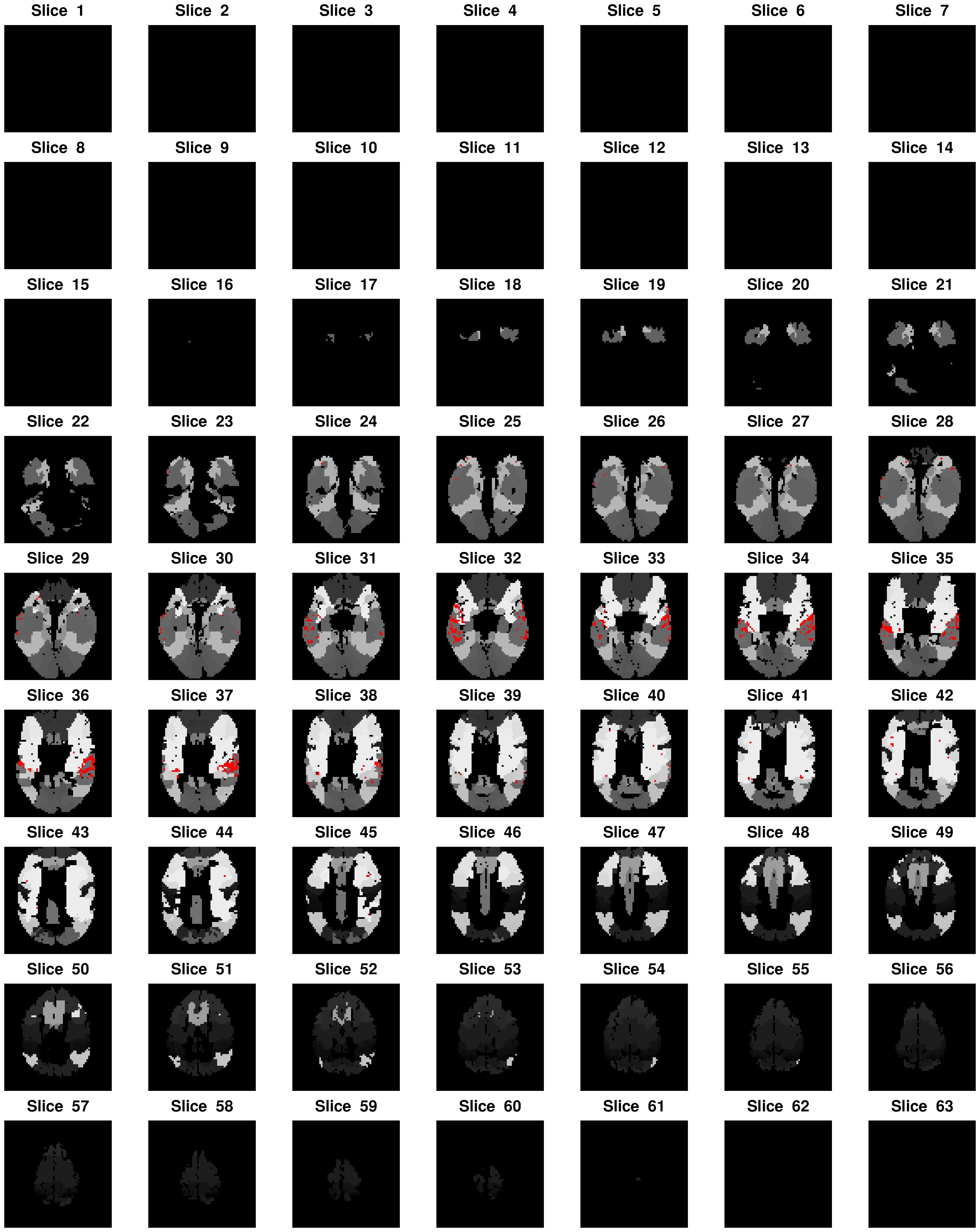

Supplement: S2 Fig — Discoveries of the proposed procedure φHO for the SPM auditory fMRI dataset on the Brodmann areas of the brain for all slices. (TIF) [file pone.0149016.s005.tif]

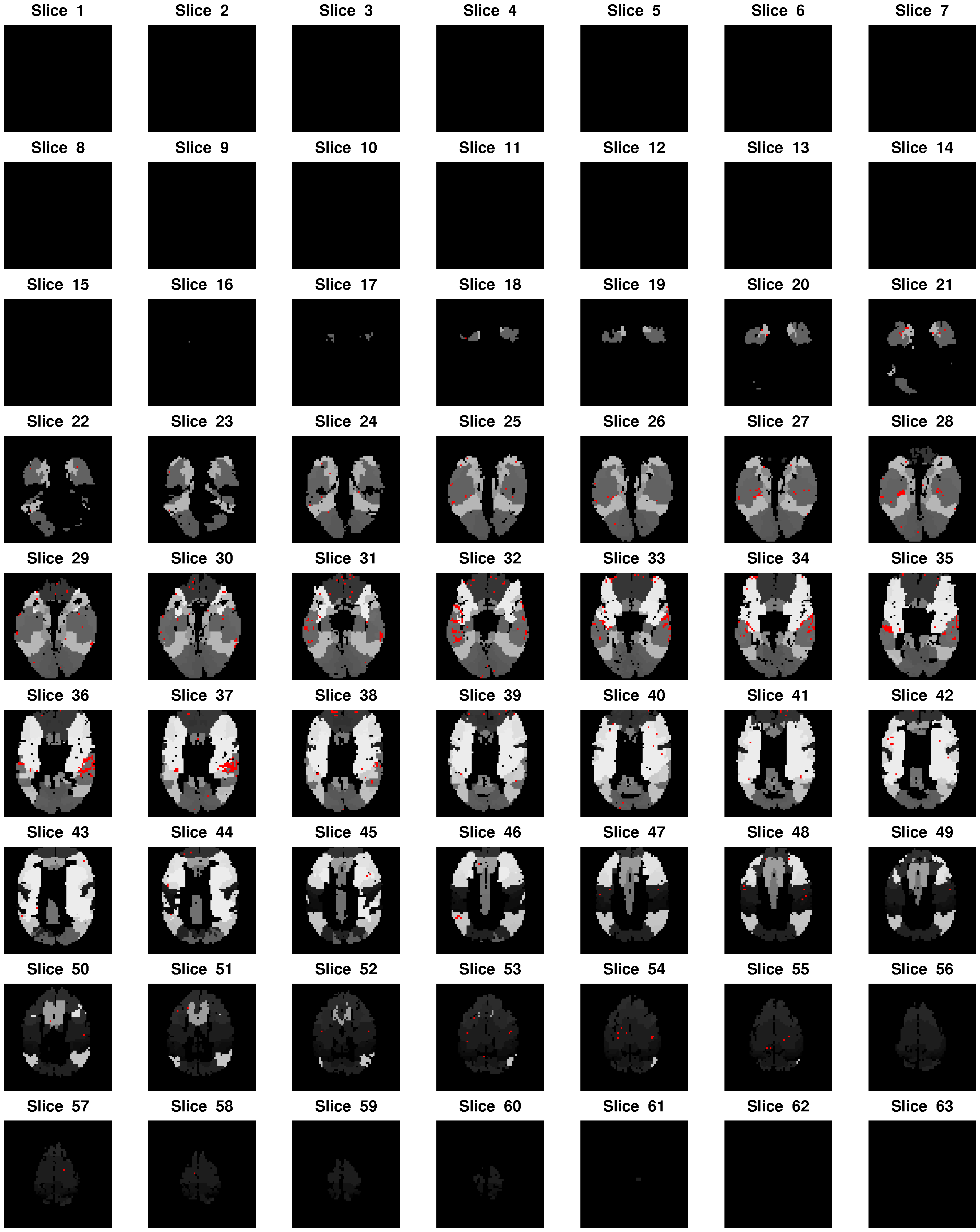

Supplement: S3 Fig — Discoveries of the procedure φBog for the SPM auditory fMRI dataset on the Brodmann areas of the brain for all slices. (TIF) [file pone.0149016.s006.tif]

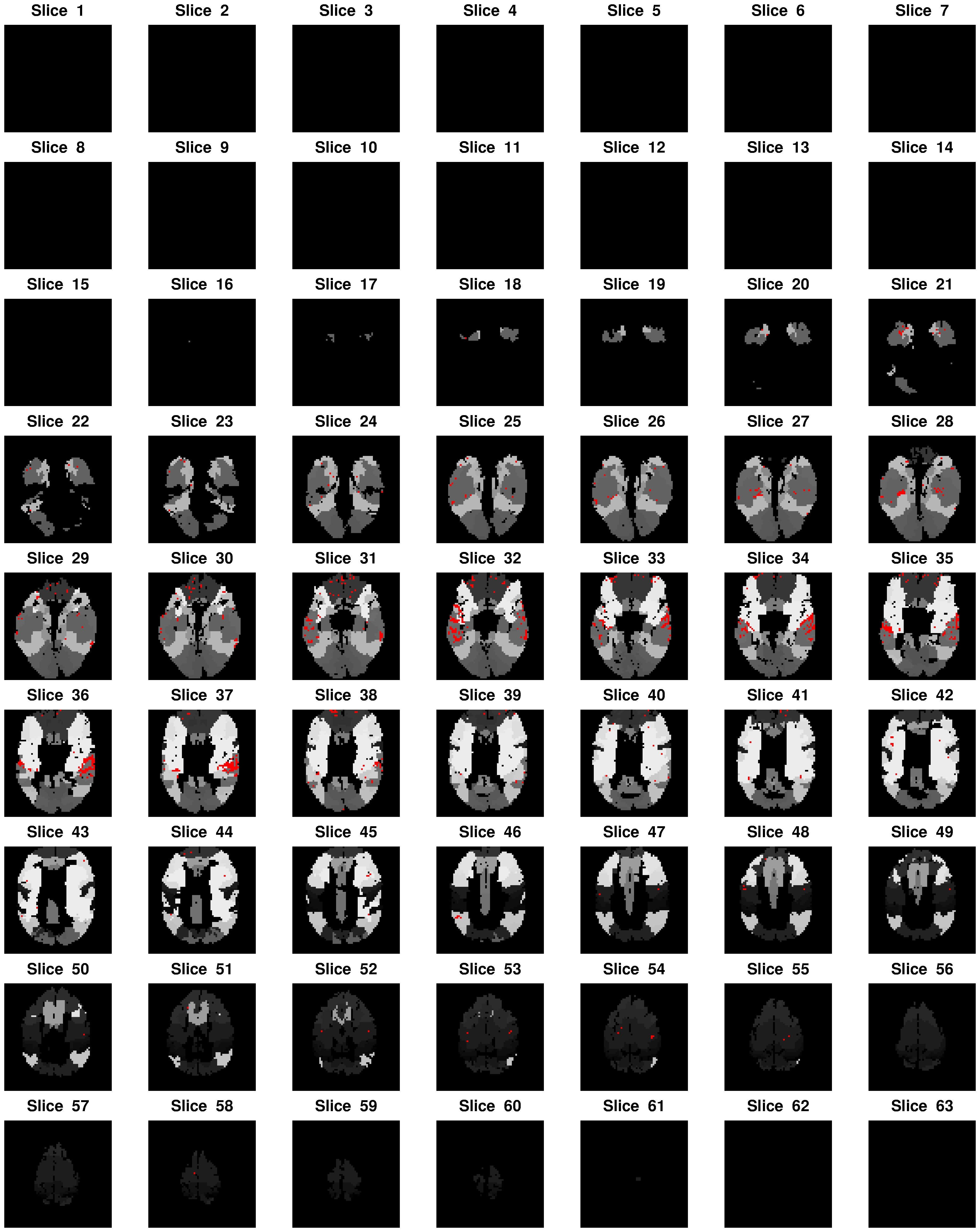

Supplement: S4 Fig — Discoveries of the procedure φLSU for the SPM auditory fMRI dataset on the Brodmann areas of the brain for all slices. (TIF) [file pone.0149016.s007.tif]

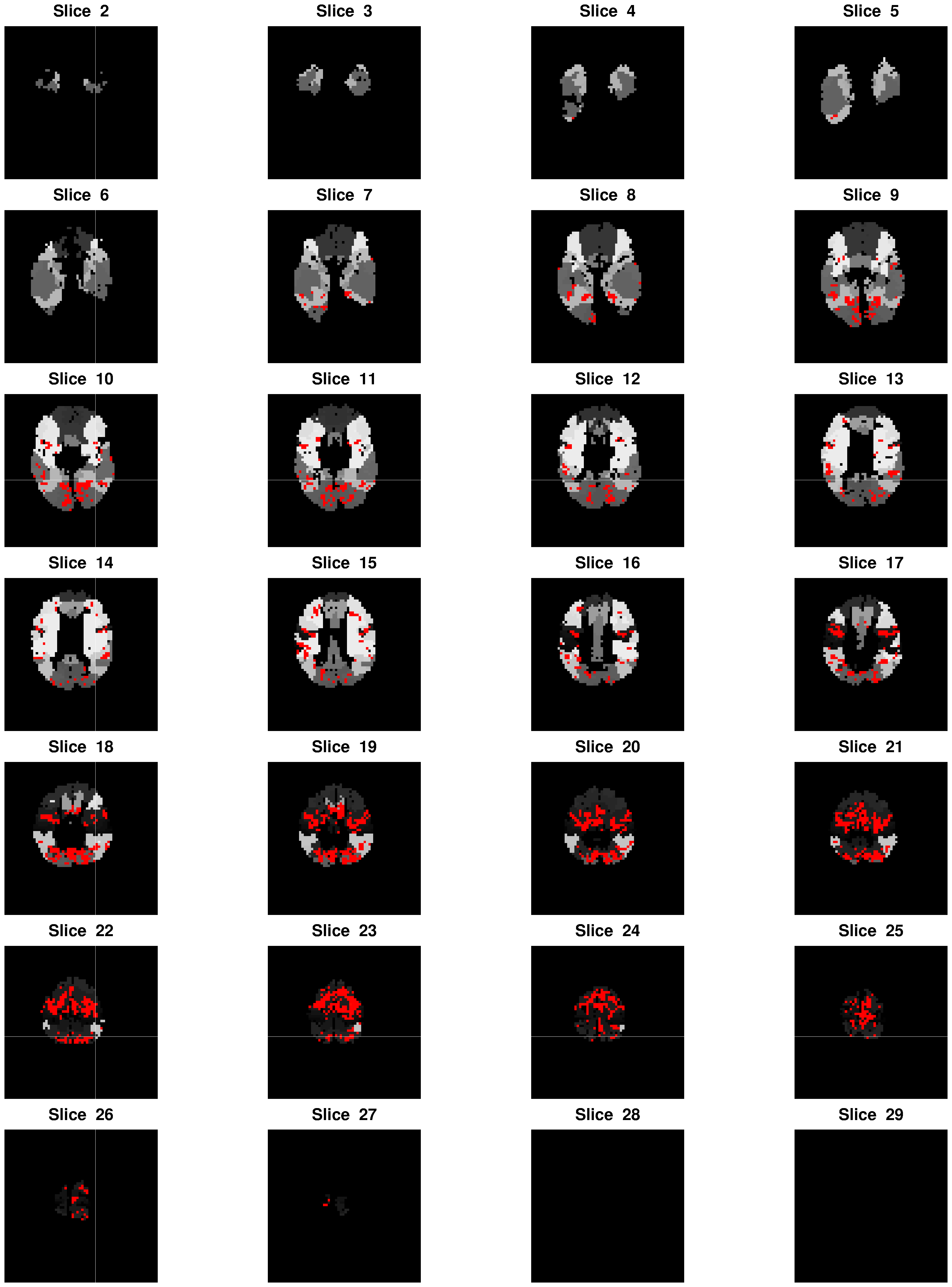

Supplement: S5 Fig — Discoveries of the proposed procedure φHO for the sports imagination task dataset on the Brodmann areas of the brain for all slices. (TIF) [file pone.0149016.s008.tif]

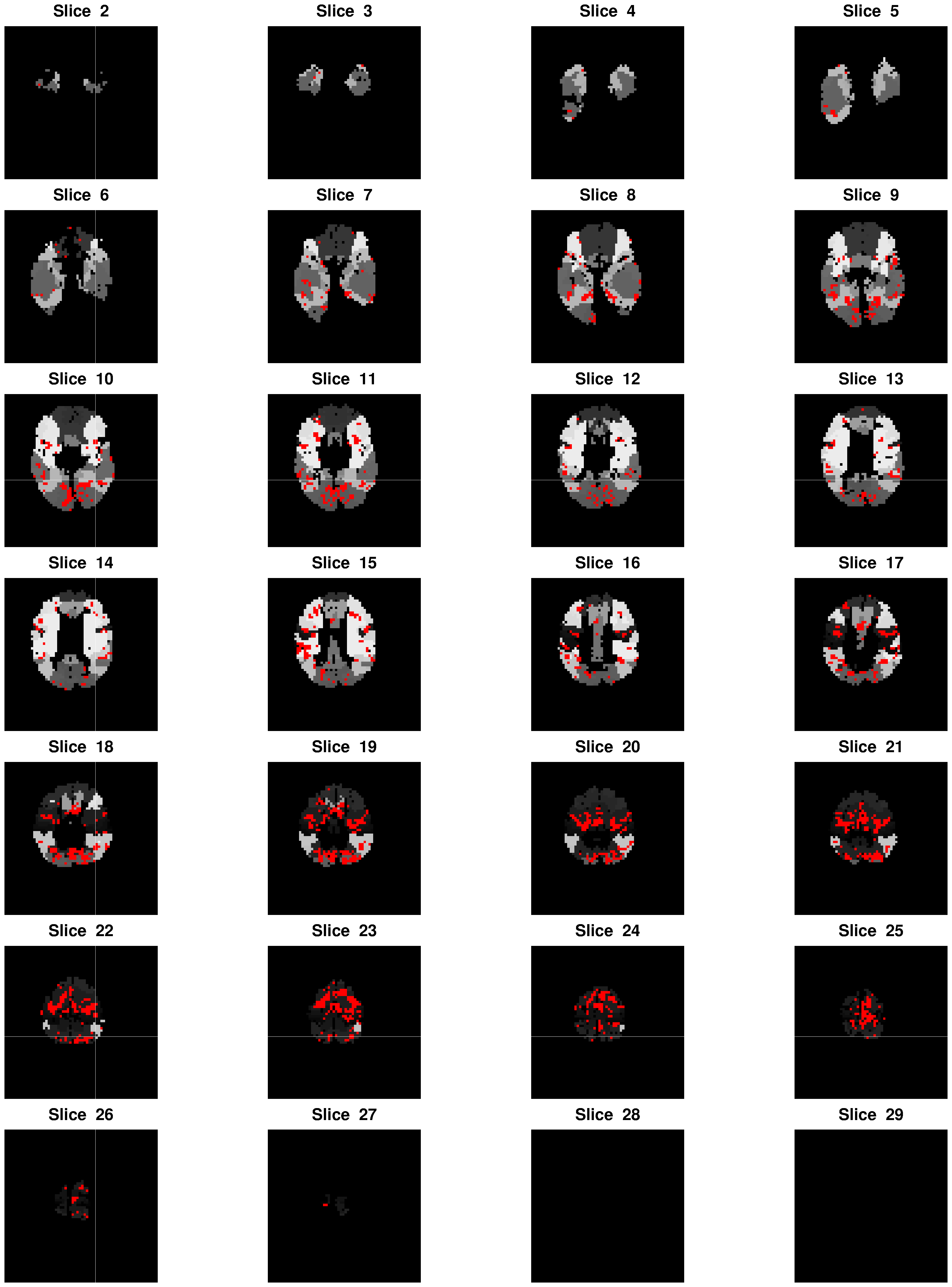

Supplement: S6 Fig — Discoveries of the procedure φBog for the sports imagination task dataset on the Brodmann areas of the brain for all slices. (TIF) [file pone.0149016.s009.tif]

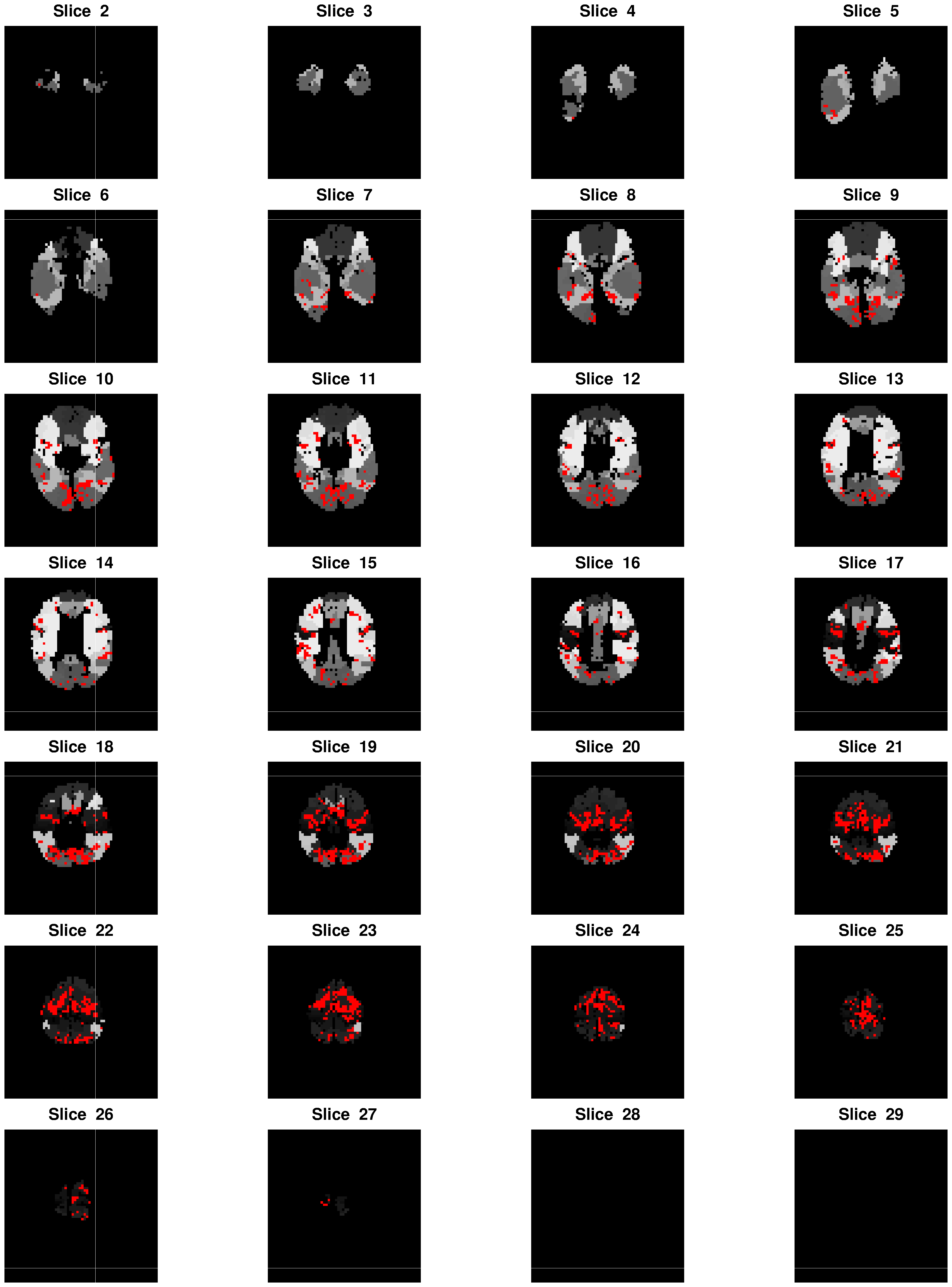

Supplement: S7 Fig — Discoveries of the procedure φLSU for the sports imagination task dataset on the Brodmann areas of the brain for all slices. (TIF) [file pone.0149016.s010.tif]
